# Baseline biomarkers of efficacy and on-treatment immune-profile changes associated with bempegaldesleukin plus nivolumab

**DOI:** 10.1038/s41698-024-00641-7

**Published:** 2024-07-19

**Authors:** Helen Gogas, Shruthi Ravimohan, Antara Datta, Aparna Chhibber, Eva Muñoz Couselo, Adi Diab, Caio Pereira, Gaëlle Quéreux, Shahneen Sandhu, Brendan Curti, Nikhil I. Khushalani, Matthew H. Taylor, Gregory A. Daniels, Anna Spreafico, Tarek Meniawy, Alfons J. M. Van Den Eertwegh, Yongliang Sun, Yull Arriaga, Ming Zhou, Georgina V. Long, Céleste Lebbé

**Affiliations:** 1 National and Kapodistrian University of Athens, Athens, Greece; 2 Bristol Myers Squibb, Princeton, NJ USA; 3 Vall d’Hebron Barcelona Hospital and Vall d’Hebron Instituto de Oncología (VHIO), Barcelona, Spain; 4 MD Anderson Cancer Center, Houston, TX USA; 5 Fundação Pio XII – Hospital de Câncer de Barretos, São Paulo, Brazil; 6 Unité Cancéro-Dermatologie, Nantes, France; 7 Peter MacCallum Cancer Centre, Melbourne, VIC Australia; 8 Eerle A. Chiles Research Institute, Providence Cancer Institute of Oregon, Portland, OR USA; 9 Moffitt Cancer Center, Tampa, FL USA; 10 Moores UCSD Cancer Center, La Jolla, CA USA; 11 Princess Margaret Cancer Centre, University Health Network, Toronto, ON Canada; 12 Department of Medical Oncology, Sir Charles Gairdner Hospital, Nedlands, Australia; 13 Department of Medical Oncology, Amsterdam UMC, VU University Medical Center, Cancer Center Amsterdam, Amsterdam, the Netherlands; 14 The Melanoma Institute Australia, The University of Sydney and Royal North Shore and Mater Hospitals, Sydney, NSW Australia; 15 Université Paris Cité, Dermato-Oncology and CIC AP-HP Hôpital Saint Louis, Cancer Institute APHP, Nord-Université Paris Cité, Paris, France; 16 INSERM U976 HIPI, Paris, France

**Keywords:** Cancer, Immunotherapy

## Abstract

In PIVOT IO 001 (NCT03635983), the combination of the investigational interleukin-2 agonist bempegaldesleukin (BEMPEG) with nivolumab (NIVO) had no added clinical benefit over NIVO monotherapy in unresectable/metastatic melanoma. Pre-defined baseline and on-treatment changes in selected biomarkers were analyzed to explore the potential mechanisms underlying the clinical observations. In each treatment arm, higher baseline tumor mutational burden or immune infiltration/inflammation was associated with improved efficacy compared with lower levels. On-treatment peripheral biomarker changes showed that BEMPEG + NIVO increased all immune cell subset counts interrogated, including regulatory T cells. This was followed by attenuation of the increase in CD8 + T cells, conventional CD4 + T cells, and systemic interferon gamma levels at later treatment cycles in the combination arm. Changes in tumor biomarkers were comparable between arms. These biomarker results help provide a better understanding of the mechanism of action of BEMPEG + NIVO and may help contextualize the clinical observations from PIVOT IO 001.

## Introduction

Over the past decade, the clinical adoption of checkpoint inhibitor immunotherapy has substantially improved outcomes in patients with metastatic melanoma^1–6^. Despite these advances, there remains a subset of patients who do not have durable responses to immunotherapy alone, resulting in an unmet need for the development of novel therapeutic strategies.

Interleukin-2 (IL-2) helps promote tumor cell death by enhancing the survival and expansion of CD4+ and CD8+ T cells as well as natural killer (NK) cells. High-dose IL-2 (HD IL-2) has been used for the treatment of metastatic melanoma^7,8^. However, its use is limited due to significant toxicities and the need for administration in an inpatient setting^7^. Bempegaldesleukin (BEMPEG), a pegylated IL-2 cytokine prodrug, was designed to activate the IL-2 pathway in a controlled and sustained fashion, with the goal of preferentially activating and expanding effector CD8+ T cells and NK cells over immunosuppressive regulatory T cells (Tregs) in the tumor microenvironment (TME)^9,10^.

Two early-phase, single-arm studies evaluated BEMPEG monotherapy^11^ or BEMPEG + nivolumab (NIVO)^12,13^ in patients with solid tumors. In the phase 1 EXCEL study (NCT02869295) in patients with advanced/metastatic solid tumors, BEMPEG monotherapy was found to increase proliferation and activation of CD4+ T cells in the peripheral blood, as well as CD8+ T cells and NK cells in the peripheral blood and TME, with a limited increase in Tregs in the TME. Although the sample size was small, BEMPEG monotherapy was observed to increase the frequency of programmed death 1 (PD-1)-positive CD8+ tumor-infiltrating lymphocytes (TILs) in the TME, supporting its use in combination with NIVO^11^. In the phase 1/2 PIVOT-02 trial (NCT02983045), the combination of BEMPEG + NIVO was found to have an acceptable safety profile, with a nonoverlapping adverse event profile, and demonstrated promising clinical activity as a first-line therapy in patients with metastatic melanoma^12,13^. These preliminary efficacy signals resulted in further exploration of clinical activity in melanoma.

On-treatment changes in biomarkers with BEMPEG + NIVO in PIVOT-02 were consistent with those seen with BEMPEG monotherapy in the EXCEL study^11,12^. Exploratory analyses of baseline tumor biomarkers in a small subset of patients in PIVOT-02 showed that higher levels of CD8+ TILs and a higher interferon gamma (IFN-γ) gene expression profiling signature were associated with greater objective response rates (ORR) and improved progression-free survival (PFS)^13^. The small exploratory analysis also showed a trend towards improved ORR and longer PFS in patients with higher (≥1%) programmed death ligand 1 (PD-L1) expression on tumor cells^13^. In addition, in an interim biomarker analysis from PIVOT-02, Hurwitz et al. found that some patients with a less favorable TME (PD-L1-low/TIL-low tumors) at baseline responded to BEMPEG + NIVO^10^. However, both EXCEL and PIVOT-02 were limited by a small sample size and the lack of a NIVO monotherapy control, which may have been insightful, given that NIVO has been shown to increase PD-L1^14,15^ and CD8 TILs^14–16^ in the TME.

Several baseline biomarkers have been associated with the efficacy of NIVO monotherapy in patients with untreated, unresectable, or metastatic melanoma. In the CheckMate 066 (NCT01721772) and CheckMate 067 (NCT01844505) phase 3 clinical trials, higher baseline tumor mutational burden (TMB) and tumor inflammation four-gene signature score were associated with increased likelihood of response to NIVO monotherapy and were predictive of longer survival^17^.

Given the observations of a relationship between baseline biomarkers and efficacy of BEMPEG + NIVO in PIVOT-02 and of NIVO monotherapy in CheckMate 066 and CheckMate 067, these biomarkers were assessed in patients enrolled in the PIVOT IO 001 study. The aim was to better understand the potential mechanisms underlying the clinical observations in this study as well as to identify biomarker-defined subgroups of patients who may benefit from BEMPEG + NIVO versus NIVO monotherapy.

In PIVOT IO 001, a randomized, phase 3 trial (NCT03635983), BEMPEG + NIVO combination therapy had no added clinical benefit over NIVO monotherapy in patients with previously untreated, unresectable, or metastatic melanoma^18^. Here, the results of biomarker analyses from PIVOT IO 001 are presented to gain insight into the lack of improvement in clinical benefit of first-line BEMPEG + NIVO over NIVO monotherapy in patients with unresectable or metastatic melanoma. This is the first study reporting comprehensive biomarker analysis from PIVOT IO 001, allowing for head-to-head comparisons between the BEMPEG + NIVO versus NIVO monotherapy treatment arms in a large patient cohort.

## Results

### Tumor biomarkers at baseline and their association with efficacy of BEMPEG + NIVO versus NIVO monotherapy

In total, 783 patients were randomized to receive BEMPEG + NIVO (*n* = 391) or NIVO monotherapy (*n* = 392). Patient characteristics and demographics, which have been published elsewhere^18^, were balanced across treatment arms (Supplementary Table 1). Patients with evaluable biomarkers were eligible for inclusion in the current analysis. The sample size of the biomarker-evaluable cohort for the intent-to-treat (ITT; *N* = 783) and ORR (all randomized patients with ≥6 months of follow-up; *N* = 543) populations is shown in Supplementary Table 2. Given the previously published exploratory correlations between select biomarkers and efficacy of BEMPEG + NIVO^12^ and NIVO monotherapy^17^, as well as the use of PD-L1 expression on tumor cells and *BRAF* mutation status as patient stratification factors in PIVOT IO 001^18^, the following biomarkers were analyzed in the present cohort: PD-L1 expression on tumor cells by immunohistochemistry (IHC), TMB by whole exome sequencing (WES), tumor inflammation (assessed by RNA sequencing [RNA-Seq] to analyze the tumor inflammation four-gene signature), levels of CD8+ TILs and forkhead box P3 (FoxP3+) cells by IHC, and *BRAF* mutation status by local testing. Baseline (pretreatment) distribution of all biomarkers assessed was balanced between treatment arms in the ITT and ORR populations (Supplementary Figure 1a, b; Table 1).

**Fig. 1:**
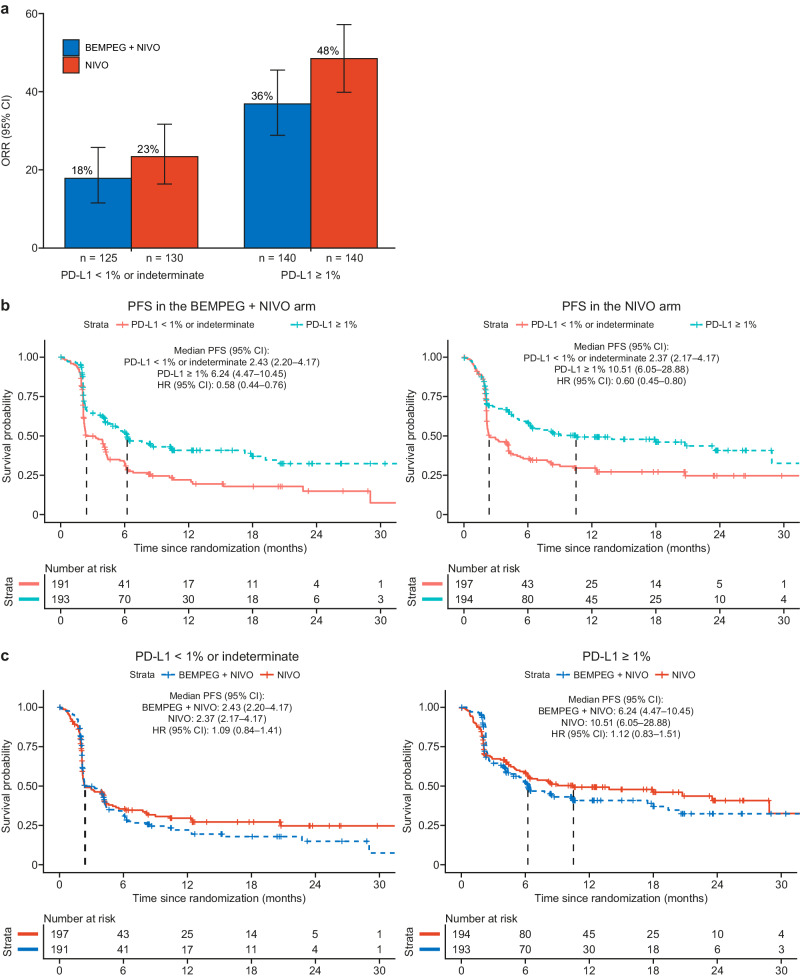
Association between pretreatment tumor PD-L1 status and efficacy of BEMPEG + NIVO vs. NIVO monotherapy. **a** ORR for patients treated with BEMPEG + NIVO vs. NIVO monotherapy, stratified by PD-L1 expression on TCs (≥1% or <1%/indeterminate); error bars represent 95% CI. **b** Kaplan–Meier curves for PFS by PD-L1 expression on TCs (≥1% or <1%/indeterminate) for patients treated with BEMPEG + NIVO or NIVO monotherapy. **c** Kaplan–Meier curves for PFS by treatment arm (BEMPEG + NIVO vs. NIVO monotherapy) for patients with PD-L1 ≥ 1% expression on TCs or <1%/indeterminate expression on TCs. BEMPEG bempegaldesleukin, CI confidence interval, HR hazard ratio, NIVO nivolumab, ORR objective response rate, PFS progression-free survival, PD-L1 programmed death ligand 1, TC tumor cell.

PD-L1 expression on tumor cells (≥1% vs. <1%/indeterminate) was a stratification factor in the PIVOT IO 001 trial^9^, so the association between tumor cell PD-L1 expression, measured at the screening visit, and efficacy in the BEMPEG + NIVO versus NIVO treatment arm was examined (see “Methods”). In both study arms, pretreatment PD-L1 levels ≥1% tended to be associated with a higher ORR compared with PD-L1 levels <1%/indeterminate. The ORR for the BEMPEG + NIVO treatment group was 36% (95% confidence interval (CI): 28–45) for PD-L1 ≥ 1% versus 18% (95% CI: 11–25) for PD-L1 < 1%/indeterminate. In the NIVO monotherapy arm, the ORR was 48% (95% CI: 39–56) for PD-L1 ≥ 1% versus 23% (95% CI: 16–31) for PD-L1 < 1%/indeterminate (Fig. 1a). Patients with higher pretreatment levels of tumor PD-L1 expression (≥1%) had longer median PFS (mPFS) than patients with PD-L1 expression <1%/indeterminate in both the BEMPEG + NIVO arm (mPFS, PD-L1 ≥ 1%: 6.24 months [95% CI: 4.47–10.45] vs. PD-L1 < 1%/indeterminate: 2.43 months [95% CI: 2.20–4.17]) and the NIVO monotherapy arm (mPFS, PD-L1 ≥ 1%: 10.51 months [95% CI: 6.05–28.88] vs. PD-L1 < 1%/indeterminate: 2.37 months [95% CI: 2.17–4.17]) (Fig. 1b). There was no added benefit of BEMPEG + NIVO over NIVO monotherapy with respect to PFS in subgroups of patients with low or high pretreatment PD-L1 expression on tumor cells (Fig. 1c).

**Fig. 2:**
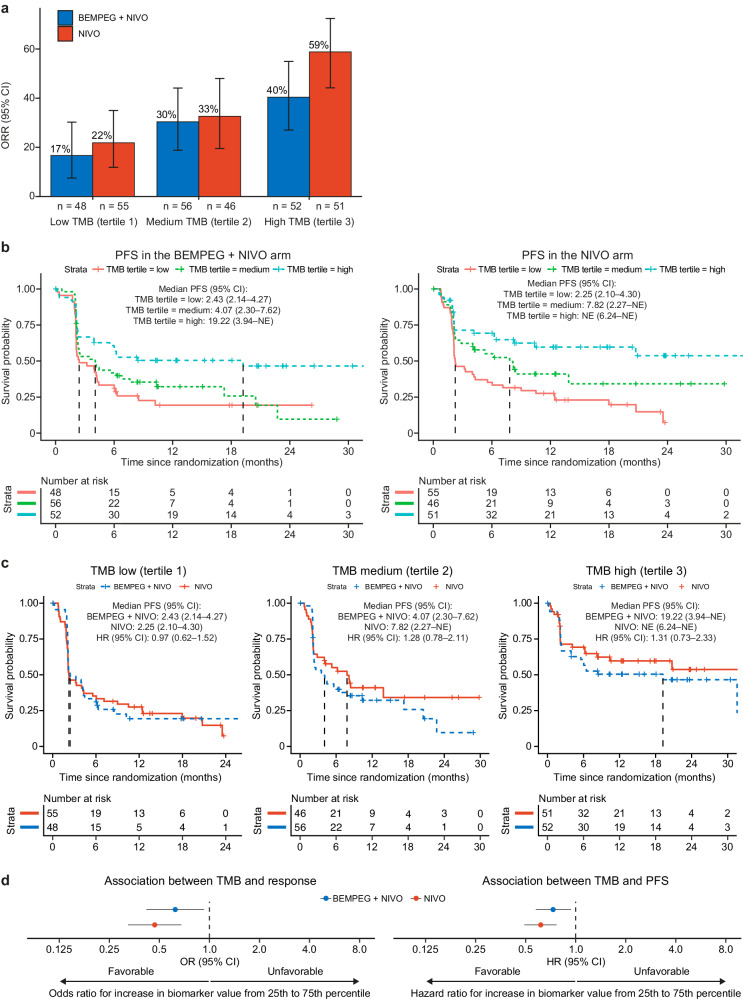
Association between pretreatment TMB and efficacy of BEMPEG + NIVO vs. NIVO monotherapy. **a** ORR for patients treated with BEMPEG + NIVO vs. NIVO monotherapy based on TMB level (low, medium, high); error bars represent 95% CI. **b** Kaplan–Meier curves for PFS by TMB (low, medium, high) for patients treated with BEMPEG + NIVO or NIVO monotherapy. **c** Kaplan–Meier curves for PFS by treatment arm (BEMPEG + NIVO vs. NIVO) for patients with low, medium, and high TMB levels. **d** Association of TMB with response and PFS; error bars represent 95% CI. TMB levels in this figure (low, medium, high) were defined based on tertiles, calculated across the complete biomarker-evaluable cohort (both arms). BEMPEG bempegaldesleukin, CI confidence interval, HR hazard ratio, NE not evaluable, NIVO nivolumab, ORR objective response rate, PFS progression-free survival, TMB tumor mutational burden.

To determine the relationship between TMB and efficacy of BEMPEG + NIVO versus NIVO, TMB was calculated from WES of patient tumor tissue taken at the screening visit (see “Methods”). Patients across both arms were grouped into TMB tertiles (Fig. 2) as well as into two groups: ≤ the median (TMB-low) and > the median (TMB-high) (Supplementary Fig. 2). Similar to PD-L1, higher pretreatment TMB levels were associated with increased likelihood of response (ORR) as well as longer mPFS than lower TMB levels in both treatment arms (Fig. 2a–d; Supplementary Fig. 2a–c). While there was no added benefit of BEMPEG + NIVO over NIVO monotherapy in any subgroup defined by TMB prior to treatment, there was a trend toward reduced clinical benefit of the combination therapy versus monotherapy in the TMB-high tertile (mPFS: 19.22 months [95% CI: 3.94–not evaluable (NE)] vs. NE [95% CI: 6.24–NE]) (Fig. 2b, c). This trend was also observed in the group with TMB > the median (mPFS: 6.8 months [95% CI: 3.94–NE] vs. 20.73 [95% CI: 6.24–NE]) (Supplementary Fig. 2b, c).

**Fig. 3:**
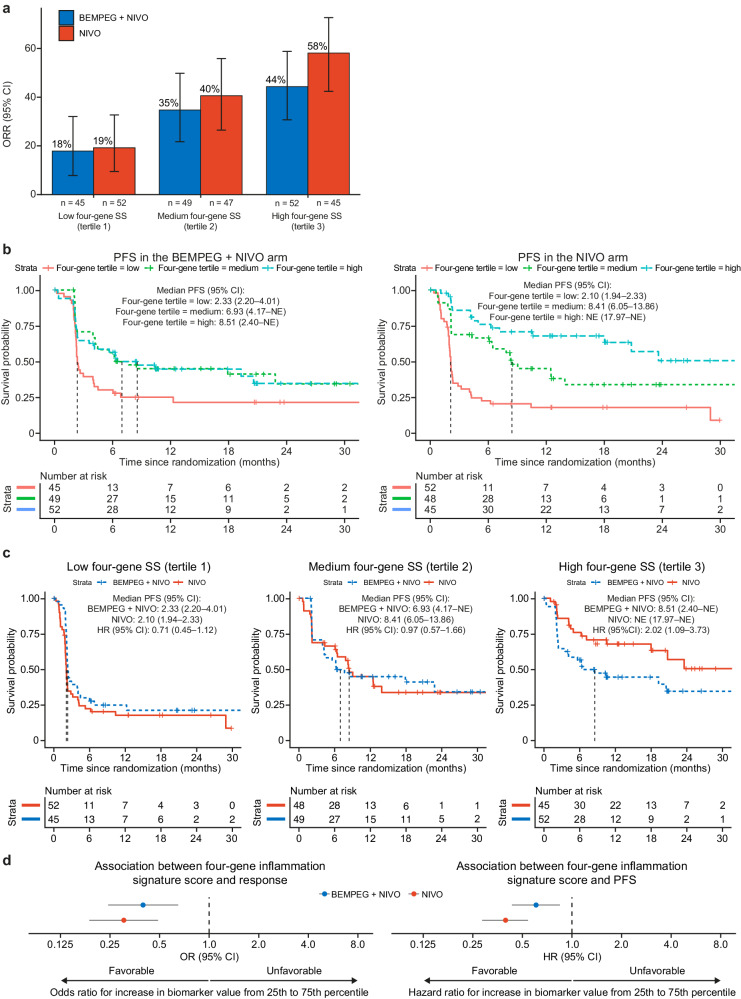
Association between pretreatment tumor inflammation four-gene signature score and efficacy of BEMPEG + NIVO vs. NIVO monotherapy. **a** ORR for patients treated with BEMPEG + NIVO vs. NIVO monotherapy, based on tumor inflammation four-gene signature score (low, medium, high); error bars represent 95% CI. **b** Kaplan–Meier curves for PFS by tumor inflammation four-gene signature score (low, medium, high) for patients treated with BEMPEG + NIVO vs. NIVO monotherapy. **c** Kaplan–Meier curves for PFS by treatment arm (BEMPEG + NIVO vs. NIVO) for patients with low, medium, and high tumor inflammation four-gene signature score. **d** Association of four-gene inflammation signature score with response and PFS; error bars represent 95% CI. Signature levels (low, medium, high) were defined based on tertiles of signature score, calculated across the complete biomarker-evaluable cohort (both treatment arms). BEMPEG bempegaldesleukin, CI confidence interval, HR hazard ratio, NE not evaluable, NIVO nivolumab, ORR objective response rate, PFS progression-free survival, SS signature score.

The association between pretreatment tumor inflammation and the efficacy of BEMPEG + NIVO versus NIVO was evaluated using a four-gene signature, comprised of the following genes: *CD274* (PD-L1), *CD8A, LAG3*, and *STAT1*^17^ (see “Methods”). The four-gene signature scores were grouped into tertiles (Fig. 3) as well as into two groups: ≤ the median (low) and > the median (high) across both arms (Supplementary Fig. 3). In both treatment arms, a higher pretreatment four-gene signature score was associated with increased likelihood of response and longer PFS (Fig. 3a–d; Supplementary Fig. 3a–c). Again, there was no added clinical benefit of BEMPEG + NIVO over NIVO monotherapy in any subgroup defined by the pretreatment four-gene signature score (Fig. 3b, c; Supplementary Fig. 3c). There was, however, a trend toward lower clinical benefit in patients treated with the combination of BEMPEG + NIVO versus NIVO monotherapy in the high inflammation subgroup (mPFS: 8.51 months [95% CI: 2.40–NE] vs. NE [95% CI: 17.97–NE]) (Fig. 3b, c). This trend was also observed in the group with four-gene signature score > the median (mPFS: 17.84 months [95% CI: 5.62–NE] vs. 23.52 [95% CI: 7.82–NE]) (Supplementary Fig. 3b, c).

In an analysis of the associations between additional pretreatment markers of tumor infiltration/inflammation and efficacy of BEMPEG + NIVO versus NIVO monotherapy, higher levels of CD8+ TILs and FoxP3+ cells in the TME, as measured by IHC (see “Methods”), were each associated with increased likelihood of response and prolonged PFS in both treatment arms (Supplementary Fig. 4a, b). Most inflammatory biomarkers evaluated at screening were positively correlated with one another (tumor inflammation four-gene signature score, CD8+ TILs, FoxP3+; PD-L1+ to a lesser extent), except for TMB (Supplementary Fig. 5). Finally, the observed association between PD-L1+, TMB, tumor inflammation four-gene signature score, CD8+ TILs, and FoxP3+ cells remained after controlling for Eastern Cooperative Oncology Group performance status, lactate dehydrogenase, presence of liver metastases, metastasis stage, sex, and age at screening (data not shown).

Lastly, the association between pretreatment *BRAF* mutation status and efficacy of BEMPEG + NIVO versus NIVO monotherapy was assessed. There was no association between pretreatment *BRAF* mutation status and efficacy in either the combination or monotherapy treatment arm (Supplementary Fig. 6).

### On-treatment changes in the peripheral blood and TME in the BEMPEG + NIVO versus NIVO monotherapy treatment arms

To determine any differences in biomarker dynamics in the peripheral blood between the BEMPEG + NIVO and NIVO monotherapy treatment arms, flow cytometry was performed on blood samples collected at baseline (Cycle [C] 1 Day [D] 1) and on-treatment (see “Methods”). Longitudinal analysis demonstrated that the combination of BEMPEG + NIVO led to initial lymphopenia on C1D3 and C5D3, and then an increased absolute lymphocyte count (ALC) at C1D8 and C5D8 relative to baseline, consistent with observations in previous studies^11,12^. These effects were not observed in the NIVO monotherapy treatment arm (Supplementary Fig. 7).

**Fig. 4:**
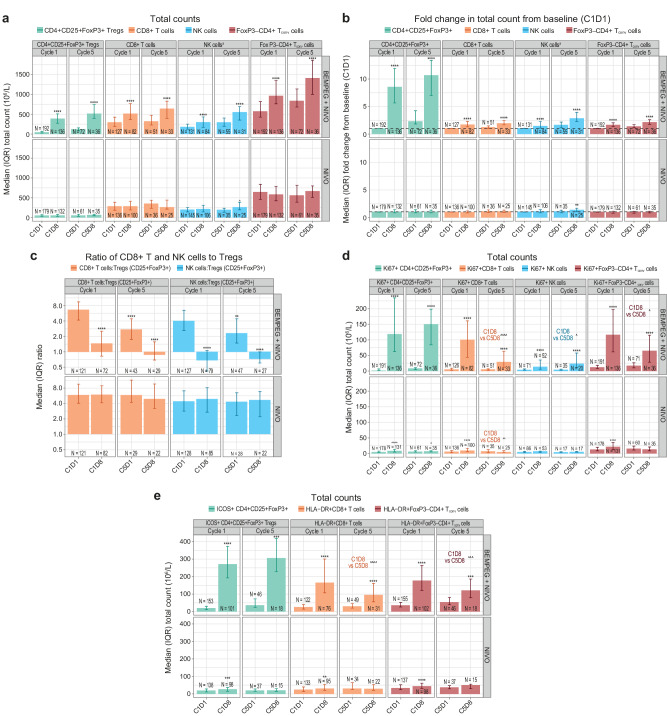
Longitudinal, on-treatment analysis of Treg, CD8+ T cells, CD4+ T_conv_, and NK cells in the peripheral blood in the BEMPEG + NIVO vs. NIVO treatment arms. **a** Longitudinal analysis of absolute CD4 + CD25+FoxP3+ Treg, CD8 + T, NK, and FoxP3 − CD4+ T_conv_ cell counts in the peripheral blood in the BEMPEG + NIVO vs. NIVO treatment arms. **b** Longitudinal analysis of fold change in CD4 + CD25+FoxP3+ Treg, CD8 + T, NK, and CD4+ T_conv_ cell counts in the peripheral blood in the BEMPEG + NIVO vs. NIVO treatment arms. **c** Longitudinal analysis of the ratio of CD8+ T cells and NK cells to Tregs in the peripheral blood in the BEMPEG + NIVO vs. NIVO treatment arms. **d** Longitudinal analysis of actively proliferating Ki67 + CD4 + CD25+FoxP3+ Treg, Ki67 + CD8+ T cells, Ki67+ NK cells, and Ki67+ FoxP3 − CD4+ T_conv_ cells in the peripheral blood in the BEMPEG + NIVO vs. NIVO treatment arms. **e** Longitudinal analysis of ICOS + CD4 + CD25+FoxP3+ Tregs, HLA-DR + CD8+ T cells, and HLA-DR+FoxP3 − CD4+ T_conv_ cells in the peripheral blood in the BEMPEG + NIVO vs. NIVO treatment arms. Error bars indicate the IQR. Asterisks indicate adjusted *P* value comparing cell counts at C1D1 with those at C1D8 or C5D8 among patients who had results available for both timepoints; ****P < 0.00001, ***P < 0.0001, **P < 0.001, *P < 0.01. Arrowheads indicate adjusted *P* value comparing counts at C1D8 with those at C5D8 among patients with results available at both timepoints; ^^^^*P* < 0.00001, ^^^*P* < 0.0001, ^^*P* < 0.001, ^*P* < 0.01. NK cells are the sum of immature (CD45+ lymph CD3−CD56hiCD16−), mature (CD45+ lymph CD3 − CD56 − CD16+), and intermediate (CD45+ lymph CD3 − CD56 + CD16+) NK cells. BEMPEG bempegaldesleukin, C cycle, D day, FoxP3 forkhead box P3, ICOS inducible costimulatory, IQR interquartile range, NIVO nivolumab, NK natural killer, T_conv_ conventional T cell, Treg regulatory T cell.

Given that peak lymphocytosis was observed on D8, changes in the individual immune cell subset counts on C1D8 and C5D8 were characterized. Compared with NIVO monotherapy, BEMPEG + NIVO resulted in substantial increases in absolute immune cell counts in the peripheral blood, including CD4 + CD25+FoxP3+ Tregs, CD8+ T cells, FoxP3−CD4+ conventional T cells (T_conv_), and NK cells on C1D8 and C5D8 (Fig. 4a). With respect to fold change from baseline, the highest magnitude of increase was observed for CD4 + CD25+FoxP3+ Tregs (~8–10-fold increase), followed by CD8+ T cells (~2-fold increase) and NK cells (~1.5–3-fold increase) on C1D8 and C5D8 (Fig. 4b). In the BEMPEG + NIVO combination arm, the ratio of CD8+ T cells to Tregs or of NK cells to Tregs decreased on C1D8, C5D1, and C5D8 compared with baseline (C1D1). By contrast, in the NIVO monotherapy arm, these ratios were maintained over time (Fig. 4c).

Changes in proliferation and activation of these immune cells were interrogated by arm. Overall, all proliferating (Ki67+) immune cell subsets investigated increased following treatment with BEMPEG + NIVO in C1 and C5 (Fig. 4d). However, when D8 changes in C5 versus C1 in the BEMPEG + NIVO arm were compared, there was an attenuation in proliferating CD4+ T_conv_ and CD8+ T cells in the peripheral blood during C5 of treatment (Fig. 4d). By contrast, levels of proliferating Tregs and NK cells were comparable between C1 and C5 (Fig. 4d). The magnitude of increase in proliferating immune cells was relatively limited in patients treated with NIVO monotherapy compared with those treated with the BEMPEG + NIVO combination (Fig. 4d).

Similar to the overall CD4 + CD25+FoxP3+ Treg counts, on-treatment increases in activated Treg cell counts (inducible costimulatory [ICOS+] Tregs) in the peripheral blood were observed at C1D8 and C5D8 in the BEMPEG + NIVO combination arm, with a comparable magnitude across the cycles (Fig. 4e). Similarly, activated (HLA-DR+) CD8+ T-cell and CD4+ T_conv_ cell counts also increased from baseline to C1D8 and C5D8 in the BEMPEG + NIVO arm (Fig. 4e). However, the magnitude of this increase was attenuated in C5 compared with C1.

**Fig. 5:**
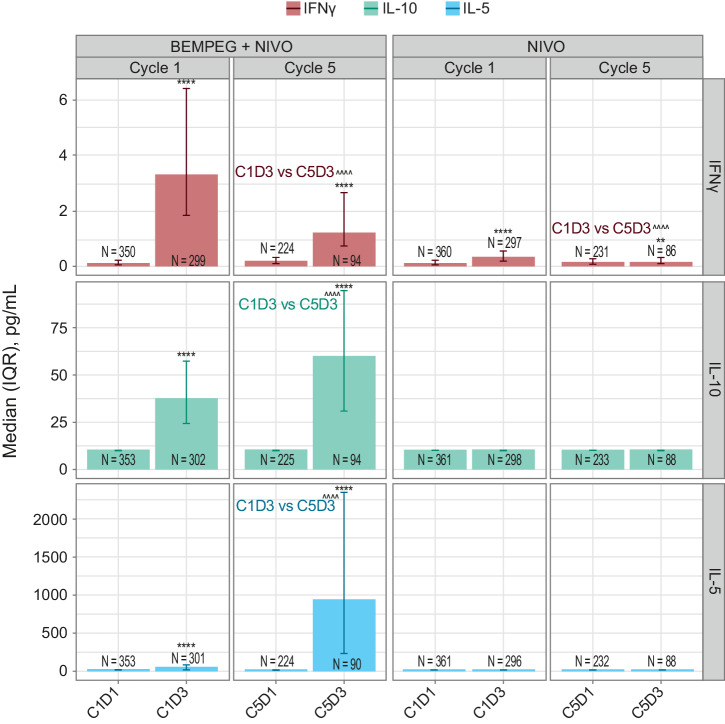
Longitudinal, on-treatment analysis of IFNγ, IL-10, and IL-5 in the peripheral blood in the BEMPEG + NIVO vs. NIVO treatment arms. Median levels of IFNγ, IL-10, and IL-5 are represented for C1D1, C1D3, C5D1, and C5D3 across treatment arms. Error bars indicate the IQR. Asterisks indicate adjusted *P* value for Wilcoxon signed-rank test comparing cell counts at C1D1 with those at C1D3 or C5D3 among patients with results available at both timepoints; ****P < 0.00001, ***P < 0.0001, **P < 0.001, *P < 0.01. Arrowheads indicate adjusted *P* values from linear mixed-effects models, shown comparing cytokine levels at C1D3 with those at C5D3; ^^^^*P* < 0.00001. For IL-10 and IL-5 in the NIVO monotherapy arm, the majority of values were below assay limit of detection at all timepoints. BEMPEG bempegaldesleukin, C cycle, D day, IFNγ interferon gamma, IL-5 interleukin-5, IL-10 interleukin-10, IQR interquartile range, NIVO nivolumab.

Changes in systemic cytokines and chemokines were also interrogated. Significant increases in systemic IFNγ were observed in the BEMPEG + NIVO treatment arm at C1D3 and C5D3 compared with C1D1 (Fig. 5). Increases in IFNγ were also observed in the NIVO monotherapy arm, but the magnitude of the increase was lower than that seen in the combination arm. Additionally, the magnitude of increase in IFNγ on C5D3 was lower than that observed on C1D3 in both treatment arms. Systemic IL-10 and IL-5 increased significantly at C1D3 and C5D3 compared with C1D1 in the BEMPEG + NIVO treatment arm. In contrast to IFNy, the magnitude of IL-10 and IL-5 increases was greater in C5 than in C1 (Fig. 5). NIVO monotherapy had minimal impact on IL-10 and IL-5 compared with combination therapy.

**Fig. 6:**
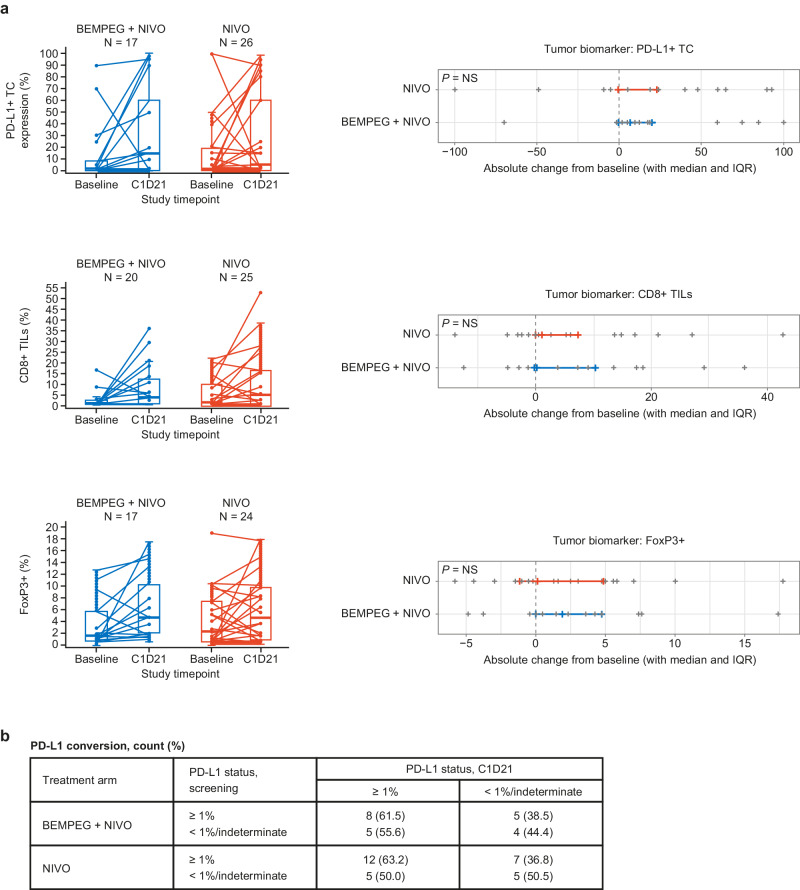
Changes in TC PD-L1+ expression, CD8+ TILs, and FoxP3+ cells in the BEMPEG + NIVO vs. NIVO treatment arms over time. **a** Absolute changes from baseline to C1D21 in % PD-L1+ TCs, % CD8+ TILs, and % FoxP3+ cells in the BEMPEG + NIVO vs. NIVO treatment arms. The difference in change from baseline to C1D21 between arms was tested using the Wilcoxon rank sum test. Box and whisker plots: center line represents median; boundaries represent 25th and 75th percentile datapoints; whiskers represent range. **b** PD-L1 conversions from baseline to C1D21 by treatment arm. Baseline, at the time of screening. BEMPEG bempegaldesleukin, C cycle, D day, FoxP3 forkhead box P3, IQR interquartile range, NIVO nivolumab, NS not significant, PD-L1 programmed death ligand 1, TC tumor cell, TIL tumor-infiltrating lymphocyte, Treg regulatory T cell.

Finally, an analysis of on-treatment changes in expression of PD-L1+ tumor cells, CD8+ TILs, and FoxP3+ cells from baseline to C1D21 showed no significant differences between treatment arms (Fig. 6a). In addition, numerically greater increases in CD8+ TILs were observed in responders versus non-responders, irrespective of treatment group (Supplementary Fig. 8). PD-L1 status at baseline versus C1D21 showed that the rate of conversion from PD-L1-negative at baseline to PD-L1-positive at C1D21 was comparable between the BEMPEG + NIVO and NIVO monotherapy treatment arms (Fig. 6b).

## Discussion

This comprehensive biomarker analysis of the large phase 3 PIVOT IO 001 trial showed that higher TMB and tumor immune infiltration/inflammation were associated with better ORR and PFS in both the BEMPEG + NIVO and NIVO monotherapy arms. This is in line with findings that TMB and inflammation are important efficacy markers for immunotherapies across a range of solid tumors, including melanoma^19–21^. However, this study revealed that no biomarker-defined subgroup of patients derived greater benefit from combination therapy than from NIVO monotherapy. Interestingly, a trend towards lower benefit of BEMPEG + NIVO was observed among biomarker-defined subgroups of patients who would otherwise have derived greatest benefit from NIVO monotherapy, such as those with inflamed tumors and immunogenic tumors, as defined by TMB. Given the proposed mechanism of action of BEMPEG, its use in combination with NIVO was hypothesized to improve the efficacy of NIVO monotherapy among patients with both favorable and unfavorable (‘cold’) TMEs^10^. It should be noted that ‘cold’ tumors have been shown to respond to anti-PD-1 therapy alone, and that on treatment biopsies may be more sensitive and specific for identifying potential responders than baseline biopsies^14,22^. Nevertheless, our study revealed that for tumors with low levels of CD8+ TILs or PD-L1+ tumor cells at screening, no differences in ORR or PFS were observed between the two treatment arms, indicating no added benefit of using BEMPEG + NIVO combination therapy over NIVO monotherapy in this subgroup. The better outcomes observed in the FoxP3 +-high subgroups may be related to generalized heightened tumor inflammation, especially given the positive correlation between higher levels of FoxP3+ cells in the TME and higher CD8+ TILs and four-gene inflammation score. Overall, the results of baseline biomarker analyses in the NIVO monotherapy arm from PIVOT IO 001 were broadly consistent with prior studies involving first-line treatment of unresectable/metastatic melanoma with NIVO monotherapy^17^.

Analysis of changes in immune cell populations in the peripheral blood during treatment indicated substantial expansion of all interrogated immune cell subsets in the BEMPEG + NIVO combination arm compared with the NIVO monotherapy arm. On-treatment shifts in the peripheral blood immune profile over time were observed in the BEMPEG + NIVO combination arm, including greater expansion of Tregs over CD8+ T cells and NK cells in later cycles as well as attenuation of activation and proliferation of T cells over time. Changes in the peripheral blood immune profile in the NIVO monotherapy arm were more limited than in the BEMPEG + NIVO combination arm. The production of cytokines with effector functions (such as IFNγ) following IL-2 receptor agonism aligns with preclinical and clinical findings^23^. Attenuation of IFNγ is also consistent with the observation of reduced T-cell proliferation/activation at later cycles in the combination arm. Furthermore, IL-10, which increased in later cycles in the combination treatment arm, can function as an immunosuppressive cytokine and be produced by various immune cells, including Tregs^24^. Despite notable on-treatment differences in the peripheral blood immune profile between treatment arms, i.e., NIVO monotherapy was associated with a lower magnitude of increase in peripheral immune cells, such as CD8+ T cells, than BEMPEG + NIVO, changes in tumor biomarkers in the TME from baseline to C1D21 were similar between treatment arms. Shifts in the peripheral blood immune profile over time, especially those observed in the BEMPEG + NIVO arm, may result in changes in TME in later cycles, but this could not be evaluated due to the lack of tumor biopsies in later treatment cycles, which was a limitation of this study.

Some of the previously reported changes in biomarkers associated with BEMPEG are in agreement with our results^12^. Specifically, PIVOT-02 showed that although higher baseline levels of CD8+ T cell tumor infiltration corresponded with a response to BEMPEG + NIVO, there was only a trend towards baseline PD-L1 expression and response to treatment^12^. On-treatment increases in ALC, CD8+ and CD4+ T cells, and NK cells in peripheral blood, as well as the proliferation and activation of these immune cell subtypes, were also noted in PIVOT-02^12^. In addition, Treg levels in peripheral blood were observed to increase^12^. In the TME, CD8+ TILs increased in response to BEMPEG + NIVO, whereas limited increases in Tregs were observed in PIVOT-02^12^. PD-L1 expression on tumors also increased in response to combination therapy in PIVOT-02. However, the PIVOT-02 study was limited by a small sample size and lack of a NIVO monotherapy control arm, precluding head-to-head comparisons. Moreover, because NIVO monotherapy has similarly been shown to increase CD8+ TILs as well as PD-L1 expression on tumors in multiple tumor types^14–16^, including melanoma^14,15^, it is difficult to determine the contribution of BEMPEG when used in combination with NIVO from the results of PIVOT-02.

Based on the observations in the present study, several hypotheses can potentially explain the lack of added clinical benefit with BEMPEG + NIVO combination therapy over NIVO monotherapy. First, combination therapy mediated substantially greater expansion of Tregs over CD8+ T cells and NK cells in the peripheral blood compared with NIVO monotherapy. This effect may be specific to BEMPEG, which may not be as selective in blocking CD25 as previously thought, and may not occur with other IL-2 receptor alpha-blocking antibodies in clinical development^25,26^, although the changes in Tregs and ALC seen with BEMPEG + NIVO are comparable with those seen with recombinant human IL-2^27^. Although previous studies did show an expansion of Tregs in the peripheral blood during treatment with BEMPEG^11,12^, the magnitude of increases was not as large as that seen in PIVOT IO 001. This may be partly explained by (a) the use of peripheral blood mononuclear cells in the previous studies rather than the use of whole blood in PIVOT IO 001, which is more appropriate for cell count measures; and (b) the use of real-time assessments with fresh blood for flow cytometry in PIVOT IO 001. Based on these observations in the peripheral blood, BEMPEG may have induced Treg-mediated suppression of T-cell proliferation and/or triggering of other negative feedback loops to achieve homeostasis. In addition, it is possible that patients may become desensitized to BEMPEG over time due to chronic stimulation and subsequent exhaustion, resulting in the observed attenuation of T-cell activation and proliferation in later cycles of combination therapy. T cells may need an off-treatment period between cytokine treatment cycles to regain their ability to expand with subsequent doses of therapy^28^. Tachyphylaxis has been seen with other interleukin agonists, such as IL-12^29^ and IL-15^30^. Considering the results from this study, the potential for tachyphylaxis should be considered when designing and evaluating the dosing schedule of other next-generation IL-2 agonists to optimize anti-tumor activity. Another possible explanation is that the dissociation of polyethylene glycol (PEG) molecules of BEMPEG from IL-2 may lead to the generation and release of a low level of unbound IL-2 over time, promoting Treg formation. However, levels of free IL-2 measured in this study followed the pharmacokinetic profile for BEMPEG active cytokine closely (data not shown), suggesting the majority of free IL-2 released from BEMPEG had a very short half-life, as expected. Furthermore, robustly expanded immune cells, such as CD8+ and NK cells, in the peripheral blood during BEMPEG + NIVO treatment may fail to appropriately traffic into the TME. This phenomenon may have implications for other IL-2 agonists. Finally, it is possible that immune cell changes in peripheral blood may have no association with clinical outcomes.

In a recent study by Hashimoto et al.^31^ that used a lymphocytic choriomeningitis virus mouse model, anti-PD-1 plus wild-type IL-2 were synergistic. However, this synergy was abrogated when anti-PD-1 was combined with alpha-blocking IL-2. This may be related to the observation that a specific subset of CD8 T cells upregulates CD25 along with CD122 and CD132, which is the high-affinity trimeric receptor. CD25 engagement with IL-2 was essential for the observed synergy between anti-PD-1 and wild-type IL-2. Based on these observations, albeit in the preclinical infectious disease setting, and those from the PIVOT IO 001 study, it is plausible that IL-2 receptor alpha-blocking strategies may inadvertently abrogate the otherwise synergistic effects of combining anti-PD-1 with wild-type IL-2.

In summary, the translational data from PIVOT IO 001, particularly the peripheral blood analysis of immune cell subsets, provide potential explanations for the lack of added clinical benefit of BEMPEG + NIVO compared with NIVO monotherapy. Results from this study should be taken into consideration and interrogated further in future studies, with the goal of attaining therapeutic synergy between IL-2 agonists and immune checkpoint inhibitors in patients with unresectable/metastatic melanoma.

## Methods

### Patients

PIVOT IO 001 is a phase 3, randomized, open-label study in patients with treatment-naive unresectable or metastatic melanoma (NCT03635983). Patients in PIVOT IO 001 were randomized 1:1 to receive the BEMPEG + NIVO combination or NIVO monotherapy and were stratified by PD-L1 expression (≥1% or <1%/indeterminate), *BRAF* mutation status (V600 mutation-positive vs. wild-type), and American Joint Committee on Cancer metastatic stage at screening. Patients were assessed for ORR and PFS by blinded independent central review per RECIST v1.1, and for overall survival, with results reported by Diab et al.^18^. The trial met regulatory requirements and was conducted in accordance with Good Clinical Practice guidelines and the Declaration of Helsinki. The study protocol was approved by independent ethics committees and the institutional review board at each participating study site, and each participant provided written informed consent. The protocol was approved by the Institutional Review Board or Independent Ethics Committee of National and Kapodistrian University of Athens, Athens, Greece; Vall d’Hebron Barcelona Hospital, Vall d’Hebron Instituto de Oncología (VHIO), Barcelona, Spain; MD Anderson Cancer Center, Houston, TX, USA; Fundação Pio XII – Hospital de Câncer de Barretos, São Paulo, Brazil; Unité Cancéro-Dermatologie, Nantes, France; Peter MacCallum Cancer Centre, Melbourne, Victoria, Australia; Eerle A. Chiles Research Institute, Providence Cancer Institute of Oregon, Portland, OR, USA; Moffitt Cancer Center, Tampa, FL, USA; Moores UCSD Cancer Center, La Jolla, CA, USA; Princess Margaret Cancer Centre, University Health Network, Toronto, Ontario, Canada; Sir Charles Gairdner Hospital, Nedlands, Australia; Amsterdam UMC, VU University Medical Center, Cancer Center Amsterdam, Amsterdam, the Netherlands; The Melanoma Institute Australia, The University of Sydney and Royal North Shore and Mater Hospitals, Sydney, New South Wales, Australia; Université Paris Cité, Dermato-Oncology and CIC AP-HP Hôpital Saint Louis, Cancer Institute APHP, Nord-Université Paris Cité F-75010 Paris, France; INSERM U976 HIPI, F-75010 Paris, France.

### Tumor biomarkers

TMB was measured using WES. Briefly, DNA extracted from pretreatment tumor tissues and matched non-tumor (whole blood) was processed using the Agilent SureSelect Human All Exon V6 in-solution hybrid capture panel (Agilent Technologies Inc., Santa Clara, CA, USA) and underwent subsequent next-generation sequencing on the Illumina NovaSeq platform (Illumina, Inc., San Diego, CA, USA). Binary alignment and map files were generated using an implementation of the genome analysis toolkit pipeline (Sentieon Inc., San Jose, CA, USA). Tumor samples were retained if total reads were >45 million, mean target coverage was >50×, and depth of coverage was >20× at 80% of the targeted capture region or higher. Normal samples were retained if total reads were >25 million, mean target coverage was >25×, and depth of coverage was >20× at 80% of the targeted capture region or higher. Somatic mutations were called by two tools: TNscope (Sentieon Inc., San Jose, CA, USA) and Strelka2 (Illumina, Inc., San Diego, CA, USA)^32^.

TMB was evaluated in patients who had sufficient WES to pass quality control from both tumor tissue and matched whole blood. Any variants that were found in a database of germline variation (gnomAD)^33^ were excluded from the TMB calculation. TMB for a subject is defined as the total number of somatic missense mutations at the target region of capture kit used for the WES assay and identified by both Strelka2 and TNscope somatic variant callers after filtering for passing variants only. For biomarker analysis, TMB levels were categorized into tertiles calculated across the complete biomarker-evaluable cohort (both arms).

The tumor inflammation four-gene signature score was derived from RNA-Seq data for four genes (*CD274* [PD-L1], *CD8A, LAG3*, *STAT1*). Briefly, RNA extracted from tumor tissues collected at screening and at C1D21 was processed using the Illumina TruSeq RNA Access in-solution hybrid capture panel (Illumina, Inc., San Diego, CA, USA) and underwent subsequent next-generation sequencing on the Illumina NovaSeq platform. RNA-Seq data were first filtered by pre-aligning with STAR^34^ (http://star.mit.edu/cluster/) to a microbial contaminant database consisting of viral, fungal, protozoan, and bacterial genomes downloaded from National Center for Biotechnology Information (NCBI) Genbank. Samples with >5% of total reads mapping to the contamination database were excluded from analysis. Reads that did not map to this contamination database were then aligned with the GRCh38 human reference genome (Ensembl 91 gene model) using STAR, and gene-level expression estimates were calculated using RNA-Seq by Expectation Maximization. Samples with <85% alignment rate were excluded from analysis. Sequencing quality was further assessed using the Picard QC tool kit (Broad Institute, Cambridge, MA, USA) and DupRadar (Bioconductor)^35^. Samples passing quality control were then used to calculate the signature score, as previously described^17^. Because RNA-Seq data were generated across multiple batches, a batch correction procedure was applied after signature score calculation.

PD-L1 expression was evaluated in tumors by IHC, as previously described^12^. Briefly, tumor samples were stained for PD-L1 using the Dako 28-8 pharmDx assay (Agilent Technologies Inc., Santa Clara, CA, USA). PD-L1 levels were defined by the percentage of positively stained tumor cells (minimum of 100 evaluable tumor cells in the sample). CD8+/Ki67 duplex IHC (Mosaic Laboratories, LCC, Lake Forest, CA, USA) was used to quantify CD8+ cells (%) as a measure of CD8+ TILs. FoxP3 singleplex IHC (Mosaic Laboratories, LCC, Lake Forest, CA, USA) was used to quantify FoxP3+ cells (%) as an approximation of Tregs in the TME. *BRAF* mutation status was defined by local testing, as previously described^12^.

### Biomarkers in the peripheral blood

Changes in biomarkers in the peripheral blood were evaluated, including FoxP3−CD4+ T_conv_, CD8+ T cells, NK cells, Treg (CD4 + CD25+FoxP3+), ICOS+ Treg, HLA-DR + CD8+ T cells, and HLA-DR+ FoxP3−CD4+ T_conv_ cells. NK cells were quantified based on the sum of immature (CD45+ lymphocytes/lymph CD3−CD56^hi^ CD16−), mature (CD45+ lymph CD3−CD56−CD16+), and intermediate (CD45+ lymph CD3 − CD56 + CD16+) NK cells. These immune subsets were assessed by flow cytometry during C1 and C5 of treatment (Supplementary Figs. 9–11). For immunophenotyping flow cytometric analysis, patient blood samples were collected in Cyto-Chex BCT tubes. After red blood cell lysis, cells were stained using fluorescently labeled antibodies specific to the respective surface markers (Supplementary Table 3). Samples were subsequently fixed, permeabilized, and stained with nuclear markers. Stained samples were analyzed on a Beckman Coulter Cytoflex S flow cytometer (BeckMan Coulter Inc., Brea, CA, USA), and the resulting data were analyzed using FlowJo v7 software (FlowJo LLC, Ashland, OR, USA) or algorithm-based automated analysis. Changes in systemic cytokines in the peripheral blood, including IFNγ (evaluated by Simoa assay; Rules-Based Medicine, Austin, TX, USA), IL-5, and IL-10 (both evaluated by multiplex Luminex assay; Rules-Based Medicine, Austin, TX, USA) were assessed during C1 and C5 of treatment. Treated patients with baseline (C1D1) and ≥1 on-treatment measurement were included in the analysis.

### Statistical analysis

All statistical analyses were conducted using R Statistical Software (version 4.0.5; R Foundation for Statistical Computing, Vienna, Austria). For analysis of biomarkers at screening and their relationship with efficacy, hazard ratios (HR), and their 95% CIs for PFS were generated by Cox proportional hazards models using the “survival” package; Kaplan–Meier curves were generated using the “survminer” package. Median survival time and 95% CIs were constructed based on a log-log transformed CI. Association with response was conducted using an ordinal logistic regression model, with response ordered as complete response (CR)/partial response (PR) > stable disease (SD) > progressive disease (PD), using the “ordinal” package.

For analysis of on-treatment changes in biomarkers, on-treatment values were compared with baseline values using the two-sided Wilcoxon signed-rank test. Where reported, the difference between arms in change from baseline was tested using the Wilcoxon rank sum test on calculated differences. On-treatment values between cycles were compared using a linear mixed effects model. *P* values shown in each figure were adjusted for multiple comparisons using the Benjamini–Hochberg procedure. *P* values are designated as follows: * or ^, *P* < 0.01; ** or ^^, *P* < 0.001; *** or ^^^, *P* < 0.0001; **** or ^^^^, *P* < 0.00001.

### Supplementary information

Supplementary material

## Data Availability

Data may be obtained from a third party and are not publicly available. Bristol Myers Squibb will honor legitimate requests for clinical trial data from qualified researchers with a clearly defined scientific objective. Data sharing requests will be considered for Phase II-IV interventional clinical trials that completed on or after January 1, 2008. In addition, primary results must have been published in peer-reviewed journals and the medicines or indications approved in the U.S., EU, and other designated markets. Sharing is also subject to protection of patient privacy and respect for the patient’s informed consent. Data considered for sharing may include non-identifiable patient-level and study-level clinical trial data, full clinical study reports, and protocols. Requests to access clinical trial data may be submitted using the enquiry form at https://vivli.org/ourmember/bristol-myers-squibb/.
