# Supplementary material for: Baseline biomarkers of efficacy and on-treatment immune-profile changes associated with bempegaldesleukin plus nivolumab
Source: NPJ Precis Oncol. 2024 Jul 19;8:150. doi: 10.1038/s41698-024-00641-7 (PMC11258232; doi:10.1038/s41698-024-00641-7)
Supplement: Supplementary file 1 — Supplementary material [file 41698_2024_641_MOESM1_ESM.pdf]

Supplementary Materials

Gogas H, *et al*. BEMPEG plus NIVO: PIVOT IO 001 biomarker analysis

Supplementary Table 1. Baseline characteristics of the ITT population[1]

Characteristics	BEMPEG + NIVO (n = 391)	NIVO (n = 392)
**Median age, years (range)**	62.0 (22–91)	61.0 (21–93)
**Sex, n (%)**		
Female	162 (41.4)	163 (41.6)
Male	229 (58.6)	229 (58.4)
**Prior adjuvant therapy, n (%)[a]**	32 (8.2)	47 (12.0)
Anti–CTLA-4 agents	4 (1.0)	6 (1.5)
Anti–PD-1 agents	9 (2.3)	12 (3.1)
BRAF inhibitors	1 (0.3)	6 (1.5)
MEK/NRAS inhibitors	1 (0.3)	6 (1.5)
Combination anti–PD-1 plus anti–CTLA-4	1 (0.3)	1 (0.3)
Combination BRAF plus MEK/NRAS inhibitors	1 (0.3)	2 (0.5)
Other investigational agents	0	1 (0.3)
Unassigned[b]	19 (4.9)	24 (6.1)
**ECOG PS, n (%)[c]**		
0	294 (75.2)	274 (69.9)
1	96 (24.6)	116 (29.6)
2	1 (0.3)	2 (0.5)
**Stratification factors, n (%)**		
Baseline PD-L1 status[d,e]		
< 1%/indeterminate	191 (48.8)	197 (50.3)
≥ 1%	193 (49.4)	194 (49.5)
BRAF mutant status[f]		
Mutant	159 (40.7)	163 (41.6)
Wild type	232 (59.3)	229 (58.4)
AJCC v8 M stage[g]		
M0/M1any[0]	265 (67.8)	256 (65.3)
M1any[1]	126 (32.2)	136 (34.7)
**Baseline LDH, n (%)**		
≤ ULN	232 (59.3)	246 (62.8)
> ULN	156 (39.9)	144 (36.7)
≤ 2 × ULN	349 (89.3)	343 (87.5)

> 2 × ULN	39 (10.0)	47 (10.0)

[a]Patients could receive more than one adjuvant therapy.

[b]Therapies that do not have an assigned category according to the data mapping dictionary (e.g., various versions of interferon).

[c]Three patients had ECOG PS 0–1 at screening but presented with ECOG PS 2 at treatment.

[d]Patients with baseline status “not reported” or “not evaluable” were not included in this table. Due to a testing site error, seven patients were reported as “indeterminate” rather than “not evaluable” in the interactive response technology system and randomized in the study.

[e]Tumor cell PD-L1 expression (≥ 1% or < 1%/indeterminate) determined using PD-L1 IHC 28-8 pharmDx (Dako, an Agilent Technologies, Inc. company, Santa Clara, CA).

[f]*BRAF* V600 mutant versus wild type.

[g]AJCC 8th edition M0/M1any[0] versus M1any[1], based on the screening imaging and laboratory test results (LDH level). Mucosal melanomas will be considered M1 for stratification.

1. Diab, A. et al. Bempegaldesleukin plus nivolumab in untreated advanced melanoma: the open-label, Phase III PIVOT IO 001 trial results. *J Clin Oncol* **41**, 4756-4767 (2023).

AJCC American Joint Committee on Cancer; *BEMPEG* bempegaldesleukin; *BRAF* v-raf murine sarcoma viral oncogene homolog B1; *CTLA-4* cytotoxic T lymphocyte antigen-4; *ECOG PS* Eastern Cooperative Oncology Group performance status; *IHC* immunohistochemistry; *LDH* lactate dehydrogenase; *M* metastatic; *MEK* mitogen-activated protein kinase; *NIVO* nivolumab; *NRAS* neuroblastoma rat sarcoma; *PD-1* programmed cell death-1; *PD-L1* programmed death ligand 1; *ULN* upper limit of normal.

Supplementary Table 2. Sample size of biomarker-evaluable cohorts

Biomarker	ITT population (N = 783) n (%) BEMPEG + NIVO, NIVO n, n	ORR population[a] (N = 543) n (%) BEMPEG + NIVO, NIVO n, n
**PD-L1 (%)**	706 (90.2)<br>349, 357	490 (90.2)<br>242, 248
**PD-L1 category[b]**	775 (99.0)<br>384, 391	536 (98.7)<br>265, 271
**TMB**	308 (39.3)<br>156, 152	308 (56.7)<br>156, 152
**Four-gene inflammatory signature**	291 (37.2)<br>146, 145	291 (53.6)<br>146, 145
**CD8+ (%)**	442 (56.4)<br>215, 227	442 (81.4)<br>215, 227
**FoxP3+ (%)**	439 (56.1)<br>215, 224	439 (80.8)<br>215, 224
***BRAF* mutation status[c]**	783 (100)<br>391, 392	543 (100)<br>271, 272

[a]The ORR population comprises all randomized patients with ≥ 6 months of follow-up. One patient in the ORR population in the NIVO arm with best overall response of “not reported” was not included in subsequent analyses.

[b]PD-L1 expression on tumor cells (≥ 1% vs. < 1%/indeterminate), per clinical database.

[c]BRAF V600 mutation status, per case report form.

BEMPEG bempegaldesleukin; *FoxP3* forkhead box P3; *ITT* intent-to-treat; *NIVO* nivolumab; *ORR* objective response rate; *PD-L1* programmed death ligand 1; *TMB* tumor mutational burden.

Supplementary Table 3. Antibodies and fluorochromes used for immunophenotyping flow cytometric analysis

FoxP3 Treg panel

Marker	Clone	Fluorochrome	Catalog	Vendor
CD45RA	HI100	BB515	564552	BD Biosciences
CD39	TU66	PerCP Cy5.5	564899	BD Biosciences
FOXP3	PCH101	APC	17-4776-42	Thermo Fisher Scientific
CD3	OKT3	AF700	317340	Biolegend
CD45	2D1	APC-H7	560178	BD Biosciences
CCR4	L291H4	BV421	359414	Biolegend
CD4	SK3	BV510	344634	Biolegend
HLA−DR	G46-6	BV650	564231	BD Biosciences
Ki67	B56	BV786	563756	BD Biosciences
PD1	MIH4	PE	12-9969-42	Thermo Fisher Scientific
CD25	CD25-4E3	PE eFluor610	61-0257-42	Thermo Fisher Scientific
ICOS	C398.4A	PE-Cy7	313520	Biolegend

T effector/memory panel

Marker	Clone	Fluorochrome	Catalog	Vendor
CD45RA	HI100	BB515	564552	BD Biosciences
Ki67	B56	PerCP-Cy5.5	561284	BD Biosciences
CD27	O323	AF647	302812	Biolegend
CD3	SK7	AF700	344822	Biolegend
CD45	2D1	APC-H7	560178	BD Biosciences
CD197 (CCR7)	GO43H7	BV421	353208	Biolegend
CD4	OKT4	BV510	300546	Biolegend
CD8	SK1	BV605	564116	BD Biosciences
HLA−DR	G46-6	BV650	564231	BD Biosciences
CD279 (PD-1)	MIH4	PE	12-9969-42	Thermo Fisher Scientific
CD152	BNI3	PE-CF594	562742	BD Biosciences
CD38	HB7	PE-Cy7	356608	Biolegend

NK panel

Marker	Clone	Fluorochrome	Catalog	Vendor
CD94	DX22	FITC	305504	Biolegend
Ki67	B56	PerCP-Cy5.5	561284	BD Biosciences
CD56	HCD56	AF647	318314	Biolegend
CD3	SK7	AF700	344822	Biolegend
CD45	2D1	APC-H7	560178	BD Biosciences
CD335 (NKp46)	9E2	BV421	564065	BD Biosciences
CD16	3G8	BV510	563830	BD Biosciences

CD27	O323	BV605	302830	Biolegend
HLA−DR	G46-6	BV650	564231	BD Biosciences
CD366 (TIM-3)	344823	PE	FAB2365P	R&D Systems
CD314 (NKG2D)	1D11	PE-CF594	562498	BD Biosciences
CD57	TB01	PE Cy-7	25-0577-42	Thermo Fisher Scientific

Supplementary Figure 1. Sample size of biomarker-evaluable cohorts and distribution by arm

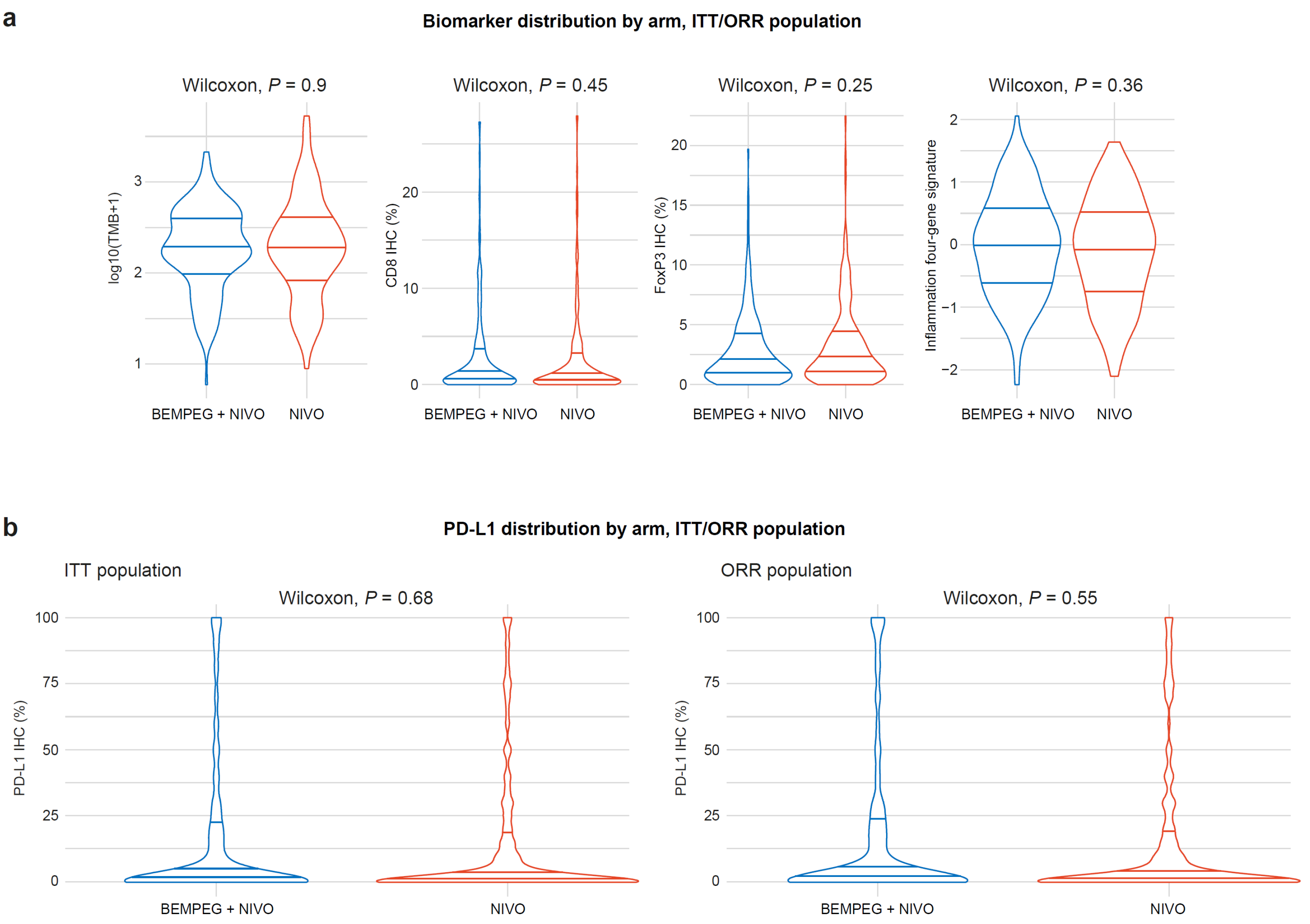

a Baseline distribution of continuous biomarkers by arm in the ITT/ORR population. Distribution of biomarkers tested was balanced between the two arms for the ITT/ORR populations. **b** PD-L1 values by arm in the ITT and ORR populations.

BEMPEG bempegaldesleukin; *IHC* immunohistochemistry; *ITT* intent-to-treat; *NIVO* nivolumab; *ORR* objective response rate; *PD-L1* programmed death ligand 1; *TMB* tumor mutational burden.

Supplementary Figure 2. Association between pretreatment TMB level (grouped by ≤ median level and > median level) and efficacy of BEMPEG + NIVO vs. NIVO monotherapy

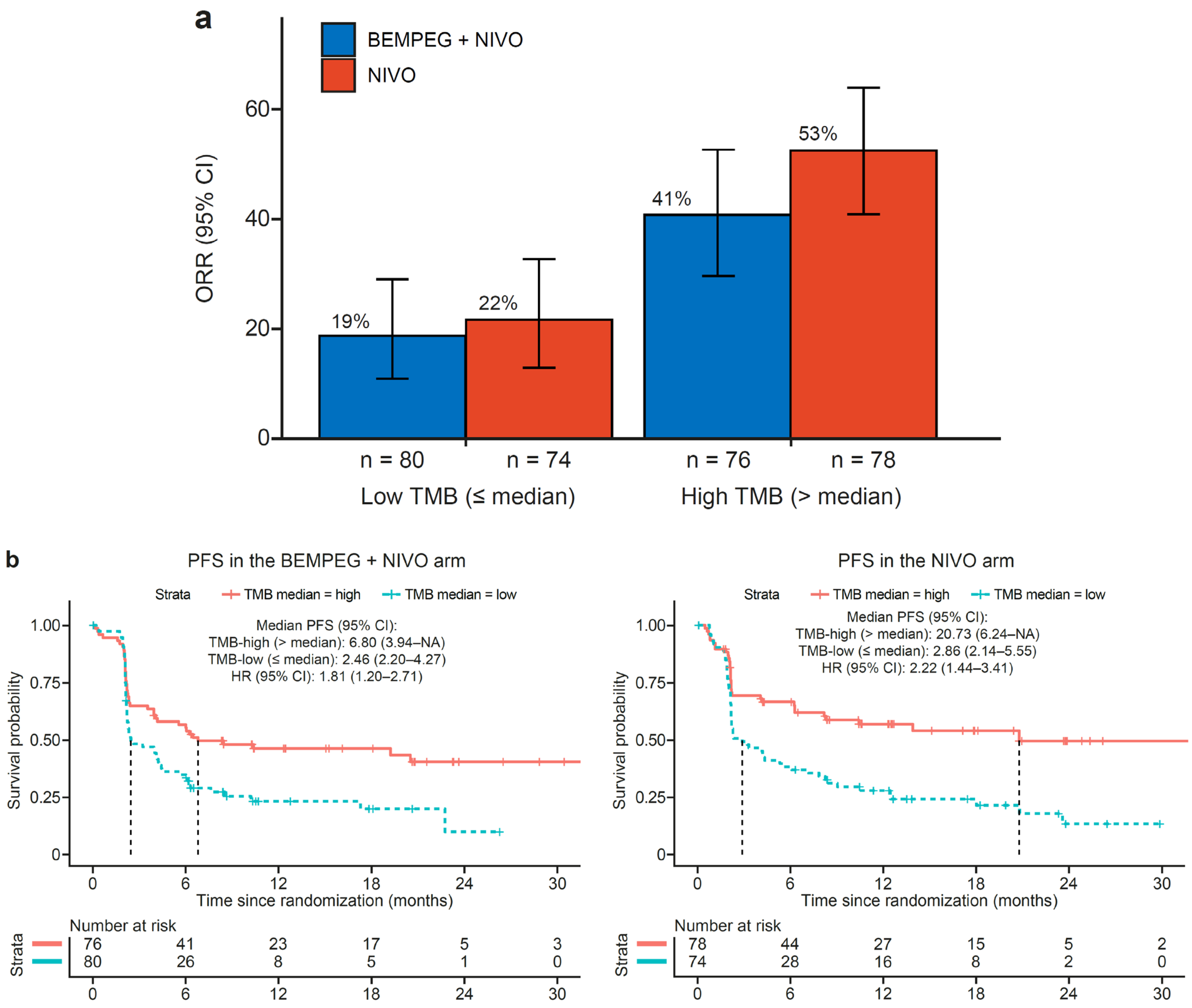

c

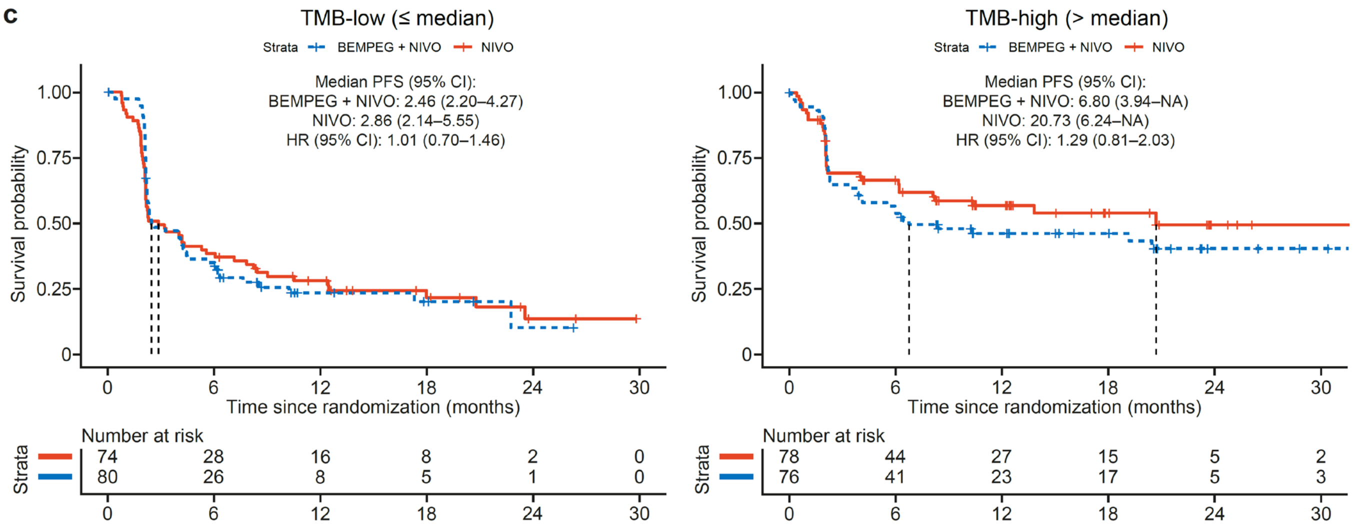

TMB levels in this figure were defined based on median TMB calculated across the complete biomarker-evaluable cohort (both arms). **a** ORR based on TMB level for patients treated with BEMPEG + NIVO vs. NIVO monotherapy; error bars represent 95% CI. **b** Kaplan–Meier curves for PFS by TMB level for patients treated with BEMPEG + NIVO or NIVO monotherapy. **c** Kaplan–Meier curves for PFS by treatment arm (BEMPEG + NIVO or NIVO monotherapy) for patients with low or high TMB.

BEMPEG bempegaldesleukin; *CI* confidence interval; *HR* hazard ratio; *NIVO* nivolumab; *ORR* objective response rate; *PFS* progression-free survival; *TMB* tumor mutational burden.

Supplementary Figure 3. Association between pretreatment tumor inflammation four-gene signature score (grouped by ≤ median level and > median level) and efficacy of BEMPEG + NIVO vs. NIVO monotherapy

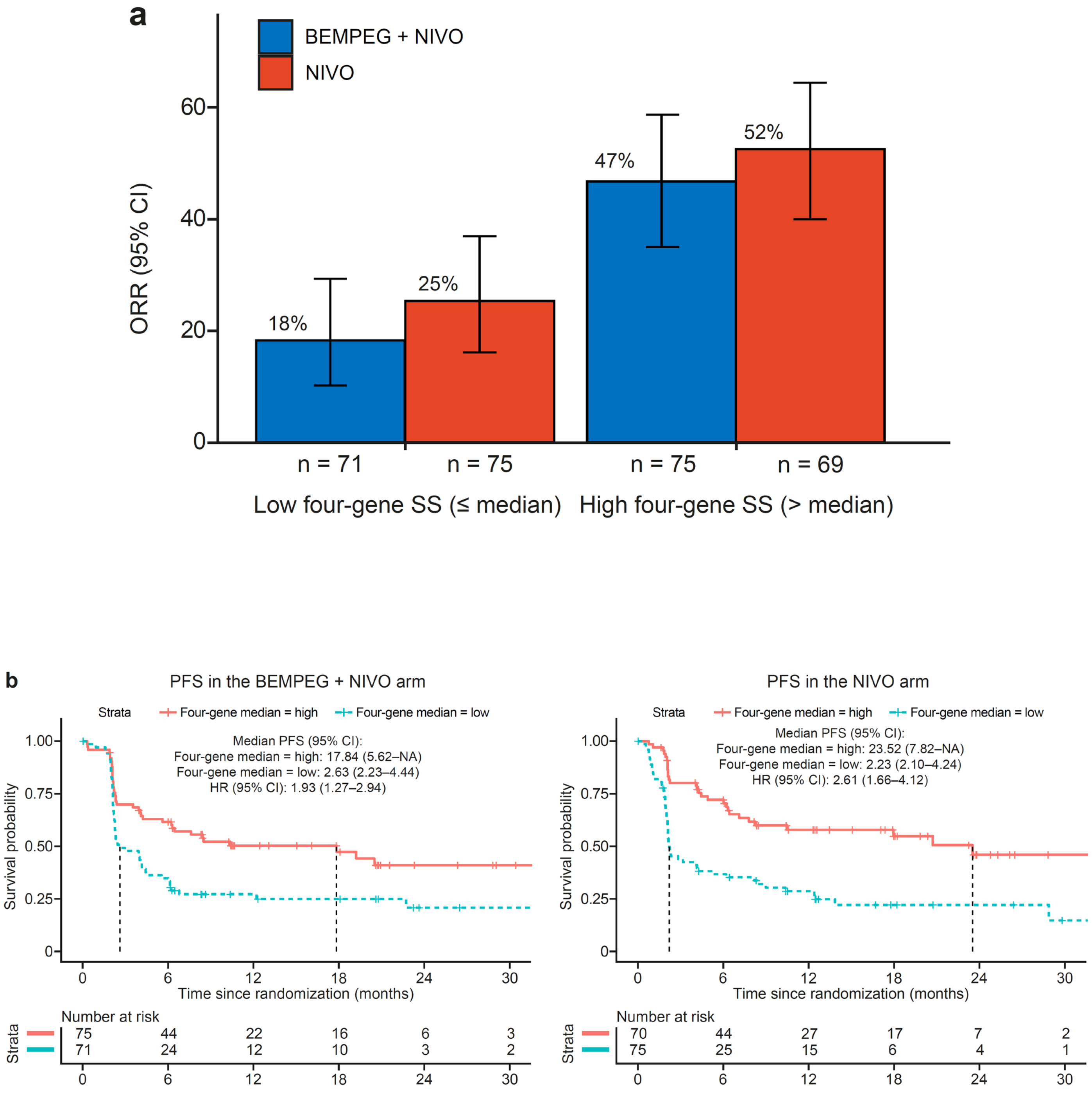

c

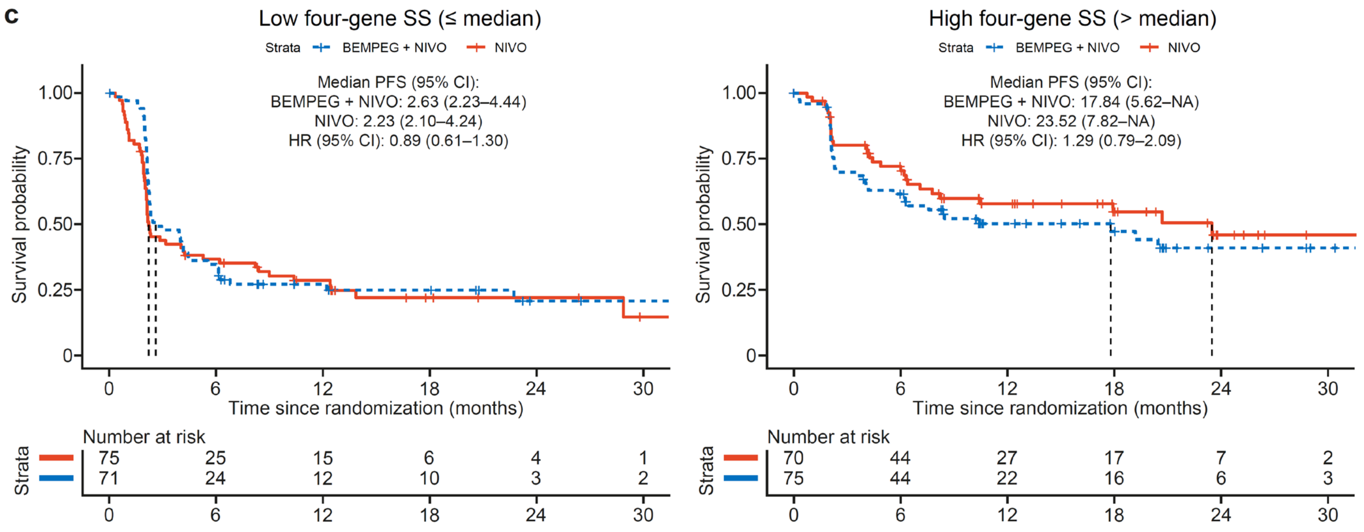

a ORR based on tumor inflammation four-gene gene signature score for patients treated with BEMPEG + NIVO vs. NIVO monotherapy; error bars represent 95% CI. **b** Kaplan–Meier curves for PFS by tumor inflammation four-gene signature score for patients treated with BEMPEG + NIVO or NIVO monotherapy. **c** Kaplan–Meier curves for PFS by treatment arm (BEMPEG + NIVO vs. NIVO monotherapy) for patients with low or high tumor inflammation four-gene signature score. Signature levels in this figure were defined based on the median signature score calculated across the complete biomarker-evaluable cohort (both arms).

BEMPEG bempegaldesleukin; *CI* confidence interval; *HR* hazard ratio; *NIVO* nivolumab; *ORR* objective response rate; *PFS* progression-free survival; SS, signature score; *TMB* tumor mutational burden.

Supplementary Figure 4. Association between pretreatment markers of tumor infiltration/inflammation a) %CD8+ TILs and b) %FoxP3+ cells in the TME and PFS, and likelihood of response in both BEMPEG + NIVO and NIVO monotherapy treatment arms

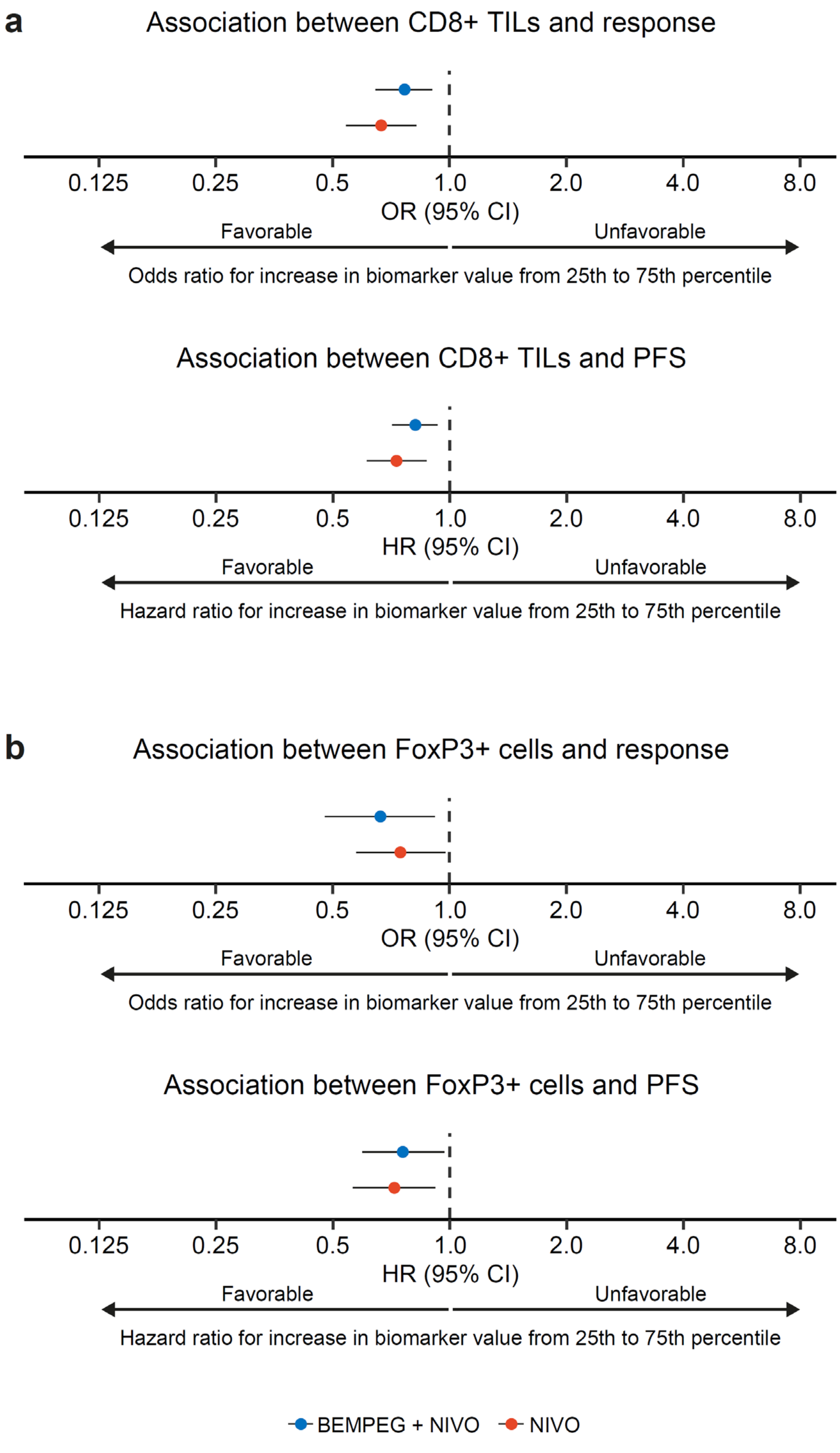

Error bars represent 95% CI.

BEMPEG bempegaldesleukin; *CI* confidence interval; *FoxP3* forkhead box P3; *HR* hazard ratio; *NIVO* nivolumab; *OR* odds ratio; *PFS* progression-free survival; *PD-L1* programmed death ligand 1; *TIL* tumor-infiltrating lymphocyte; *TMB* tumor mutational burden.

Supplementary Figure 5. Heatmap depicting Spearman correlation between markers of tumor infiltration/inflammation at screening

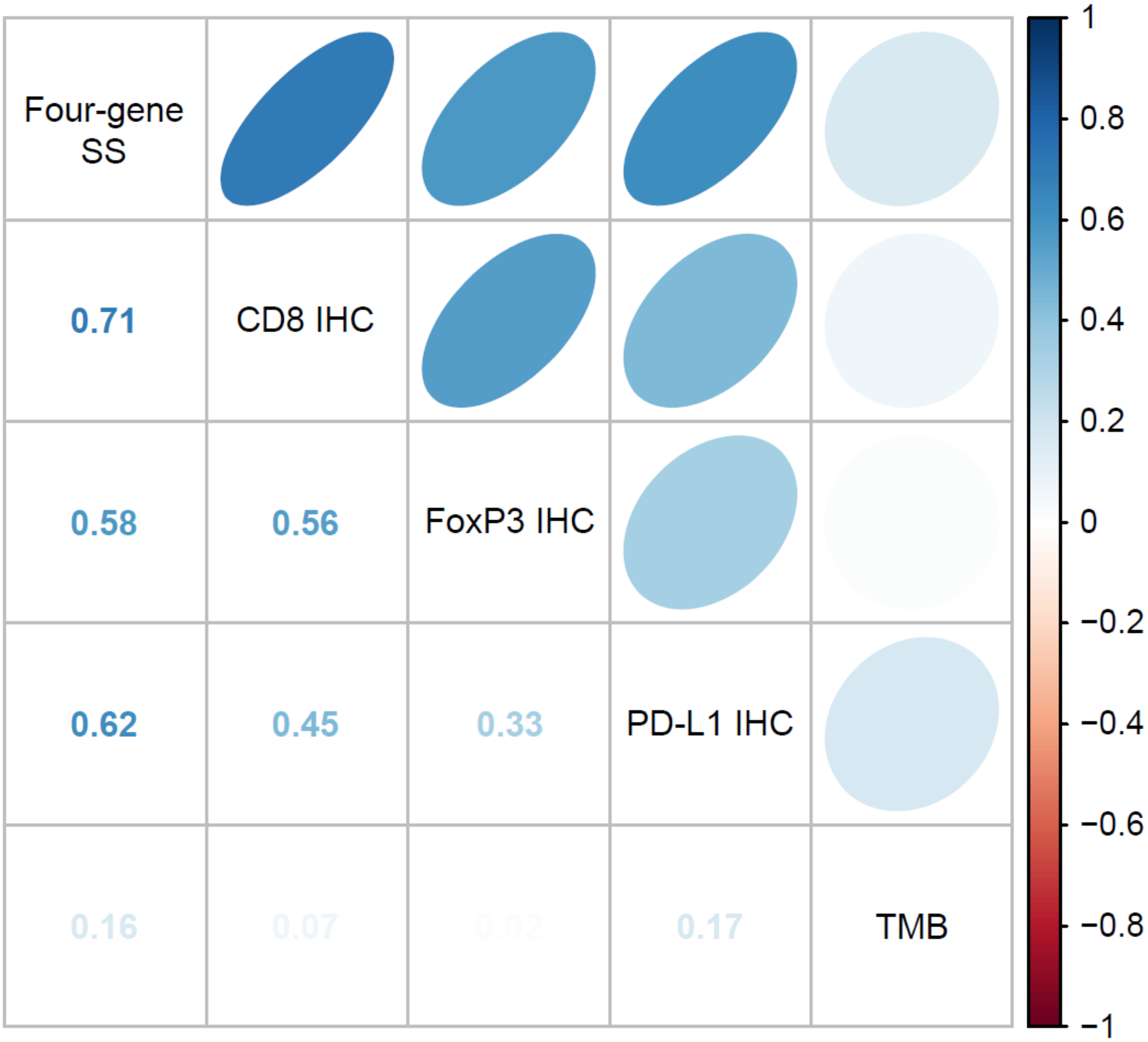

IHC immunohistochemistry; *PD-L1* programmed death ligand 1; *SS* signature score; *TMB* tumor mutational burden.

Supplementary Figure 6. Association between BRAF mutation status and efficacy of BEMPEG + NIVO vs. NIVO monotherapy

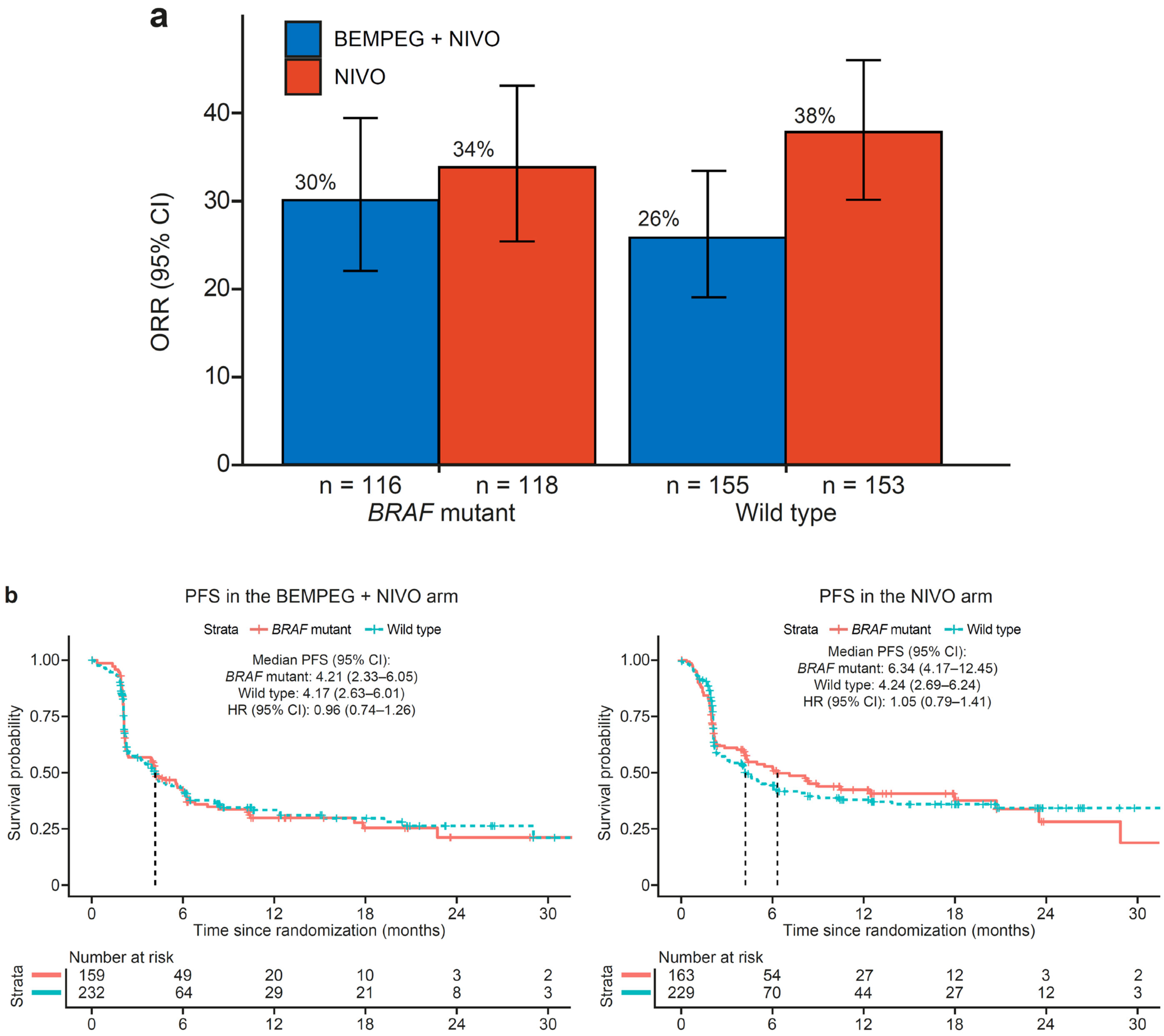

c

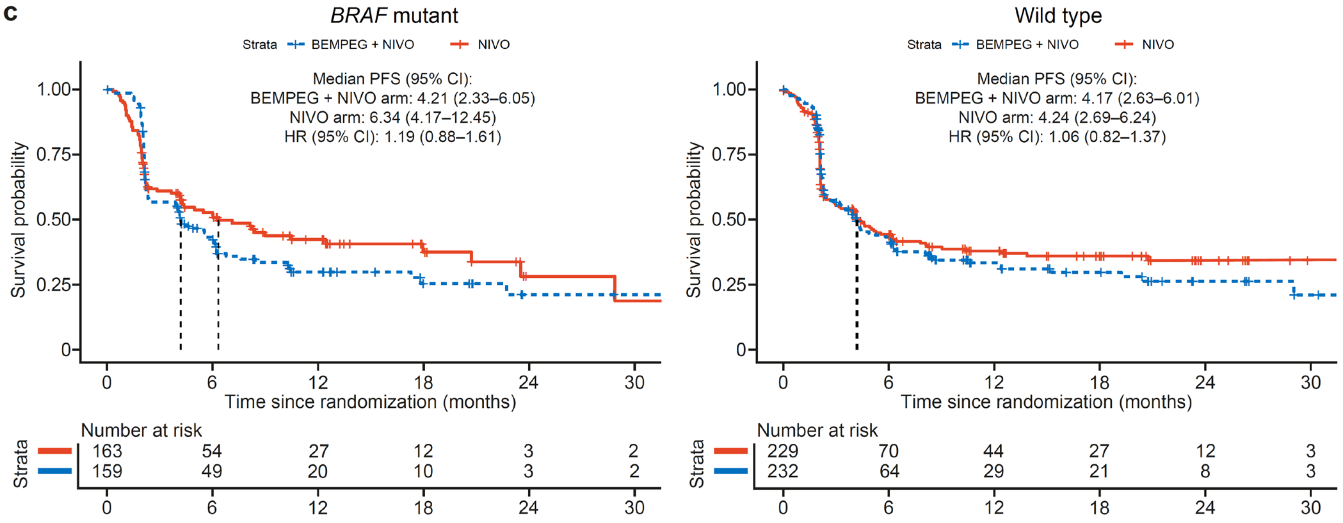

a ORR represented for BEMPEG + NIVO vs. NIVO monotherapy, stratified by tumor *BRAF* V600 status; error bars represent 95% CI. **b** Kaplan–Meier curves for PFS by tumor *BRAF* mutation status for patients treated with BEMPEG + NIVO or NIVO monotherapy. **c** Kaplan–Meier curves for PFS by treatment arm (NIVO vs. BEMPEG + NIVO) for patients with *BRAF* V600 wild type and mutant tumors.

BEMPEG bempegaldesleukin; *CI* confidence interval; *HR*, hazard ratio; *NIVO*, nivolumab; *ORR* objective response rate; *PFS* progression-free survival; *PD-L1* programmed death ligand 1.

Supplementary Figure 7. ALC increases in response to BEMPEG + NIVO combination treatment

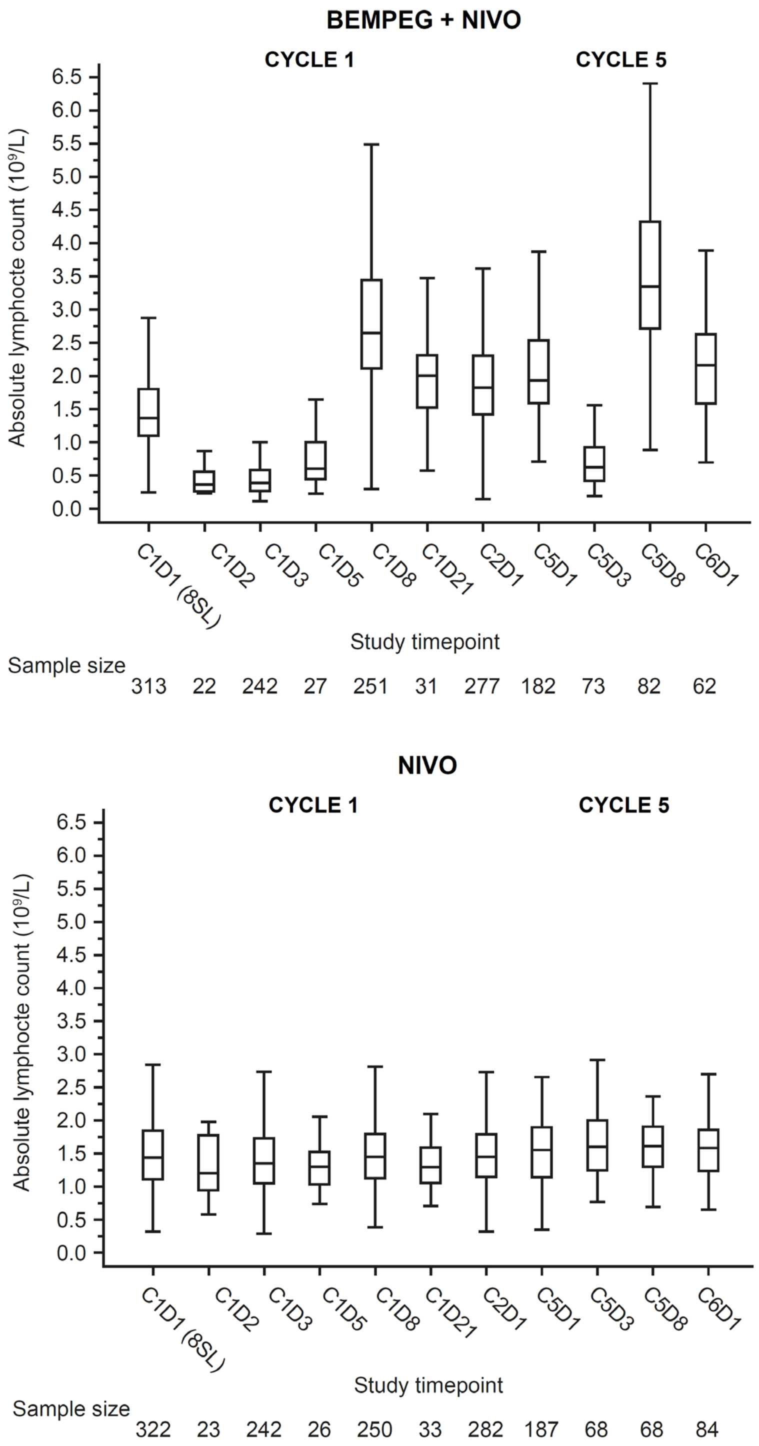

Longitudinal analysis of ALC cell count in BEMPEG + NIVO vs. NIVO monotherapy treatment arms. Each box shows the IQR and the line represents the median value. Whiskers represent maximum and minimum values.

ALC absolute lymphocyte count; *BEMPEG* bempegaldesleukin; *C* cycle; *D* day; *IQR* interquartile range; *NIVO* nivolumab.

Supplementary Figure 8. Changes in PD-L1+ expression on tumor cells, CD8+ TILs, and FoxP3+ cells from baseline to C1D21 in the BEMPEG + NIVO and NIVO monotherapy arms, grouped according to response

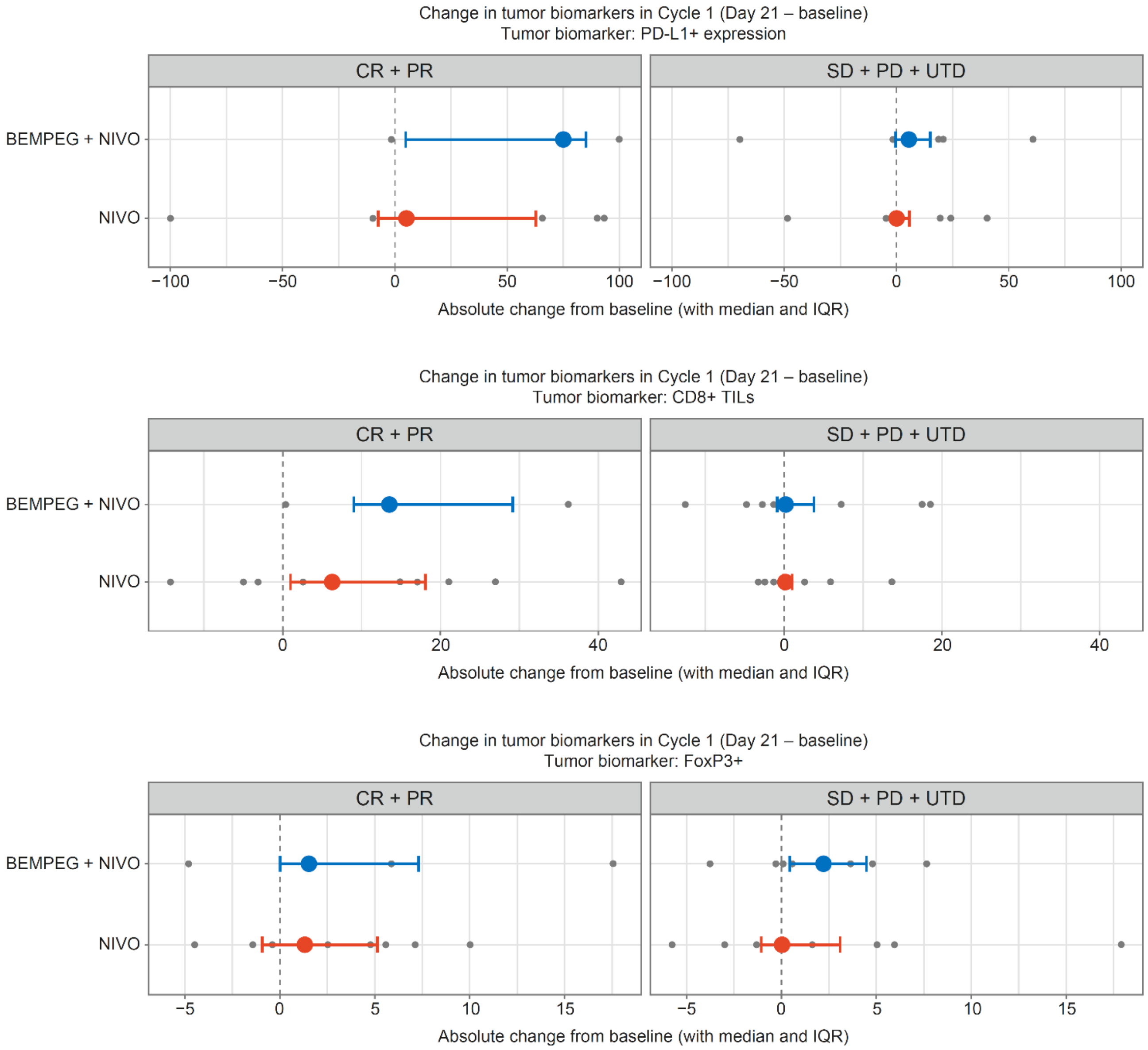

Error bars represent the IQR.

BEMPEG bempegaldesleukin*; CR* complete response*; FOXP3* forkhead box P3*; IQR* interquartile range*;* *NIVO* nivolumab; *PD* progressive disease*; PD-L1* programmed death ligand 1*; PR* partial response*;* *SD* stable disease*; TIL* tumor-infiltrating lymphocyte; *UTD* unable to determine.

Supplementary Figure 9. Gating strategy for Treg flow cytometry panel

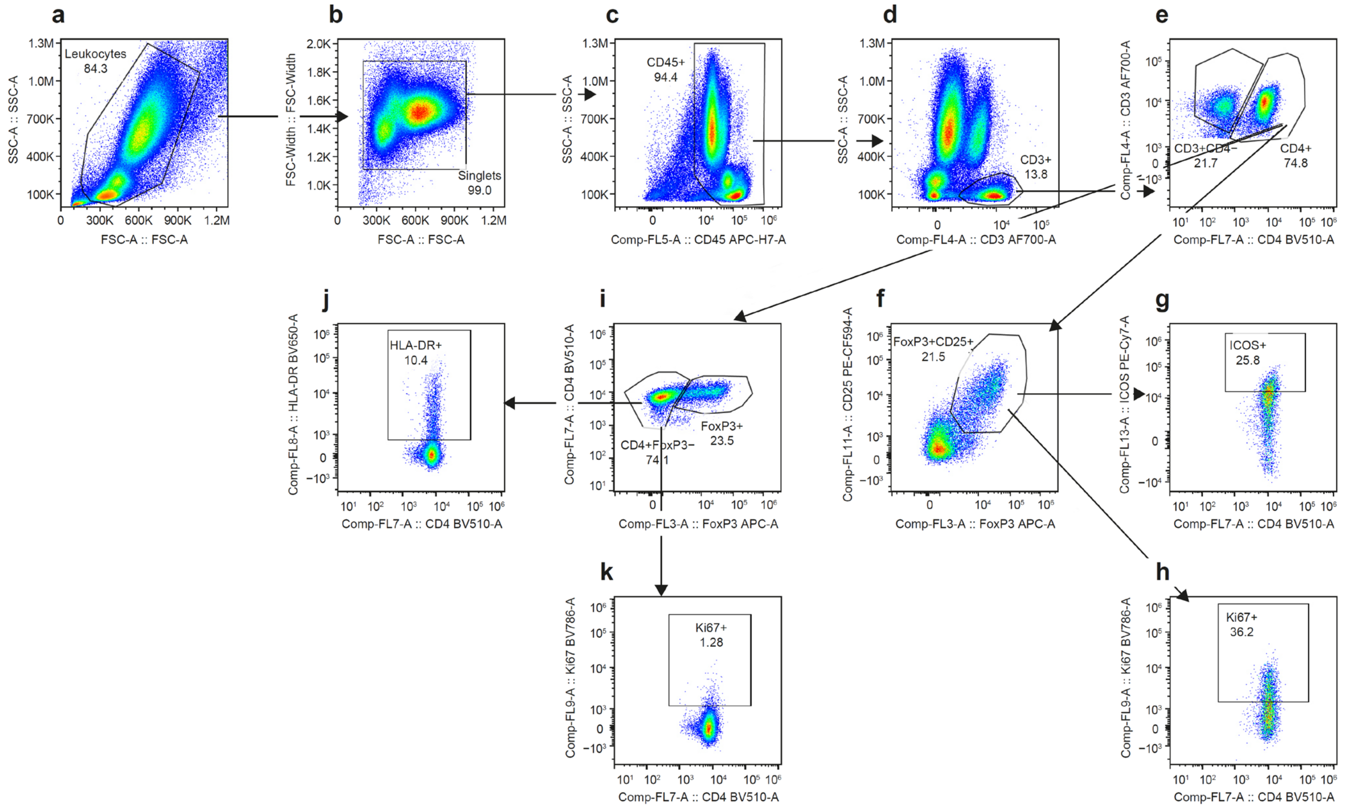

Leukocytes **(a)** were defined by FSC-A vs. SSC-A, followed by singlets **(b)** using FSC-A vs. FSC-W. CD45+ cells **(c)** were gated by CD45+ vs. SSC-A. CD3+ vs. SSC-A was applied to gate CD3+ T cells **(d)**. CD4+ T cells **(e)** were defined. From CD4+ T cells, FoxP3+CD25+ Tregs **(f)** were defined by FoxP3 vs. CD25 and subsequently subdivided into ICOS+ Tregs **(g)** and Ki67+ Tregs **(h)**. CD4+FoxP3-conventional T cells **(i)** were gated and further subdivided as HLA−DR+ CD4 conventional **(j)** and Ki67+ CD4 conventional T cells **(k)**.

Supplementary Figure 10. Gating strategy for Teff/mem flow cytometry panel

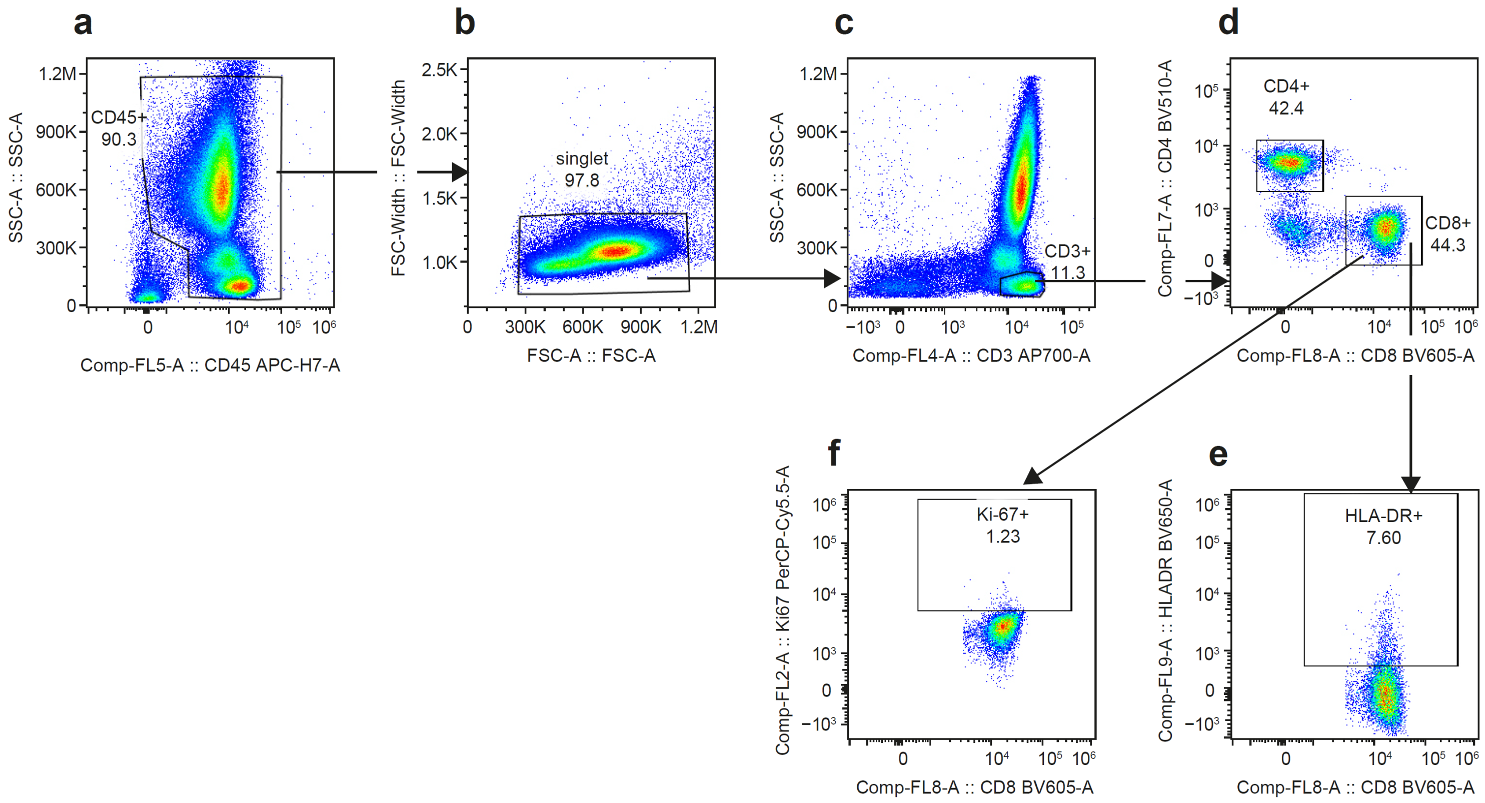

CD45+ leukocytes **(a)** were gated using CD45 vs. SSC-A, followed by singlets **(b)** by FSC-A vs. SSC-W. CD3+ T cells **(c)** were defined. From the CD4 vs. CD8 dot plot, CD8+ T cells **(d)** were gated. CD8+ T cells were further subdivided as HLA−DR+ CD8 cells **(e)** and Ki67+ CD8 cells **(f)**.

Supplementary Figure 11. Gating strategy for NK flow cytometry panel

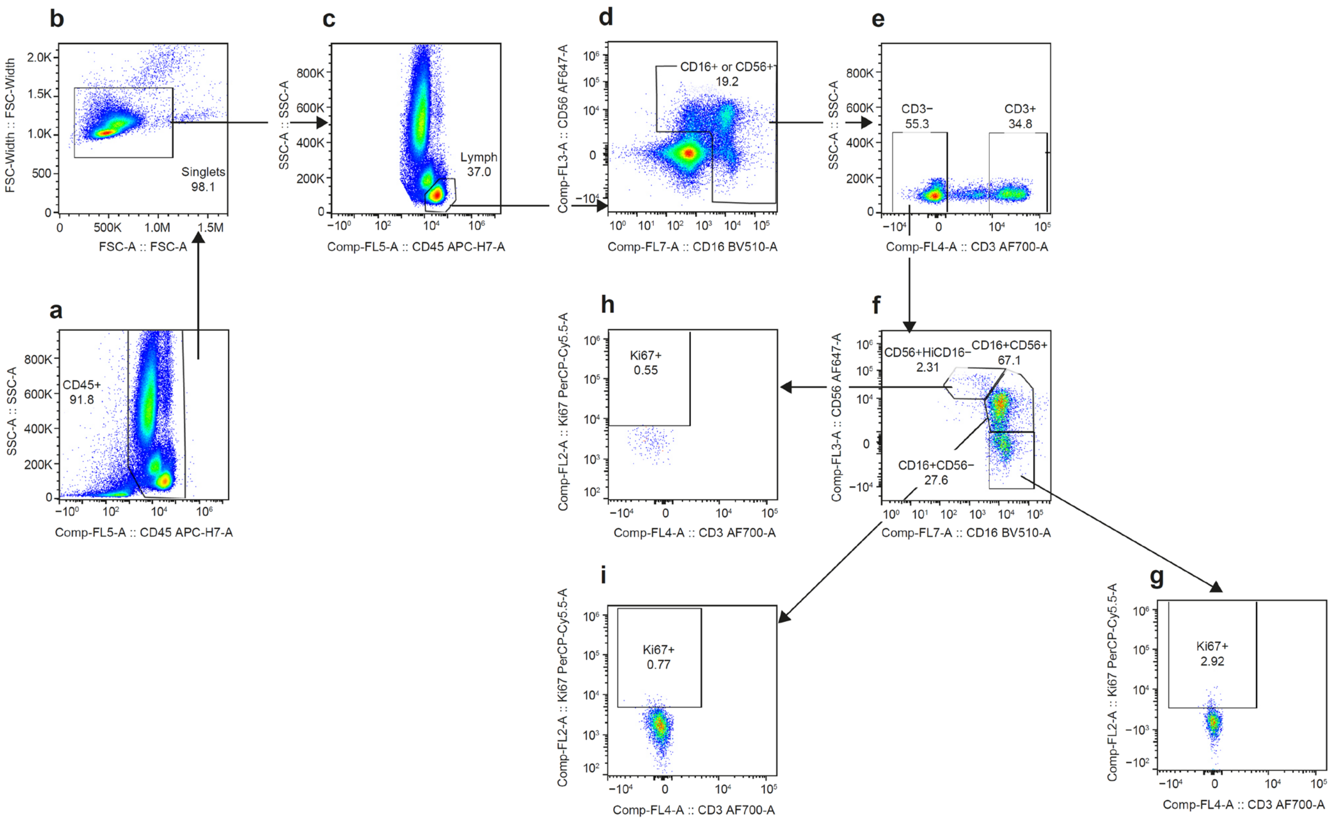

CD45+ leukocytes **(a)** were gated using CD45 vs. SSC-A, followed by singlets **(b)** by FSC-A vs. FSC-W. Lymphocytes **(c)** were defined by CD45 vs. SSC-A. Total NK cells **(d)** were gated by either CD16+ or CD56+. Total NK cells were subdivided to CD3+ NK **(e)**. From the CD16 vs. CD56 dot plot, CD3− NK cells were further subdivided to CD56+HiCD16−, CD16+CD56+, and CD16+CD56− subsets **(f)**, with respective Ki67+CD56+HiCD16− **(h)**, Ki67+CD16+CD56+ **(i)**, and Ki67+CD16+CD56− **(g)**.
